# Remodeling and destabilization of chromosome 1 pericentromeric heterochromatin by SSX proteins

**DOI:** 10.1093/nar/gkz396

**Published:** 2019-05-22

**Authors:** Sofie Traynor, Niels Erik Møllegaard, Mikkel G Jørgensen, Nadine H Brückmann, Christina B Pedersen, Mikkel G Terp, Simone Johansen, Jerome Dejardin, Henrik J Ditzel, Morten F Gjerstorff

**Affiliations:** 1 Department of Cancer and Inflammation Research, Institute for Molecular Medicine, University of Southern Denmark, J.B. Winsløws Vej 25, DK-5000 Odense, Denmark; 2 Department of Cellular and Molecular Medicine, University of Copenhagen DK-2200, Denmark; 3 Department of Biochemistry and Molecular Biology, Institute for Natural Sciences, University of Southern Denmark, Campusvej 55, DK-5000 Odense, Denmark; 4 Institute of Human Genetics CNRS-Université de Montpellier UMR 9002.141 rue de la Cardonille, 34000 Montpellier, France; 5 Department of Oncology, Odense University Hospital, Sdr. Boulevard 29, DK-5000 Odense, Denmark; 6 Academy of Geriatric Cancer Research (AgeCare), Odense University Hospital, Sdr. Boulevard 29, DK-5000, Denmark

## Abstract

Rearrangement of the 1q12 pericentromeric heterochromatin and subsequent amplification of the 1q arm is commonly associated with cancer development and progression and may result from epigenetic deregulation. In many premalignant and malignant cells, loss of 1q12 satellite DNA methylation causes the deposition of polycomb factors and formation of large polycomb aggregates referred to as polycomb bodies. Here, we show that SSX proteins can destabilize 1q12 pericentromeric heterochromatin in melanoma cells when it is present in the context of polycomb bodies. We found that SSX proteins deplete polycomb bodies and promote the unfolding and derepression of 1q12 heterochromatin during replication. This further leads to segregation abnormalities during anaphase and generation of micronuclei. The structural rearrangement of 1q12 pericentromeric heterochromatin triggered by SSX2 is associated with loss of polycomb factors, but is not mediated by diminished polycomb repression. Instead, our studies suggest a direct effect of SSX proteins facilitated though a DNA/chromatin binding, zinc finger-like domain and a KRAB-like domain that may recruit chromatin modifiers or activate satellite transcription. Our results demonstrate a novel mechanism for generation of 1q12-associated genomic instability in cancer cells.

## INTRODUCTION

Genome instability is a hallmark of cancer and plays a key role in tumor initiation and progression (1). Thus, understanding the mechanisms of this instability is crucial for better cancer diagnostics and treatment. The chromosome 1q12 region contains the largest heterochromatin site in the genome, comprising a megabase stretch of satellite II and III DNA repeats. This pericentromeric heterochromatin (PCH) structure is prone to breakage and the resulting translocations and duplications of the 1q arm are among the most frequent genetic aberrations in cancer (2). Importantly, gain of 1q material has been linked to the pathogenesis of multiple malignancies (3–7) and correlates with poor clinical outcome (8–10). 1q duplications may contribute to tumor development by increasing the dosage of cancer driver genes (11). This is exemplified in breast cancer, where a region of the 1q arm that encodes genes that support a cancer stem cell phenotype is amplified in 10–30% of primary tumors and 70% of recurring tumors (12).

Why 1q12 PCH is prone to rearrangements remains unclear. This region contains fragile sites that could predispose to breakage, but the initiating factor may be loss of normal epigenetic control of chromatin structure. Repetitive satellite DNA is generally kept in a constitutively repressed heterochromatic state, which is established by SUV39H1/2-mediated methylation of H3K9 and recruitment of heterochromatin protein 1 (HP1) (13,14). HP1 implements chromatin repression by interacting with other epigenetic factors and marks, including DNA methylation (14). Importantly, hypomethylation of PCH satellite DNA is a common event in cancer and may perturb normal control of chromatin structure (15–20). The importance of DNA methylation in epigenetic regulation of PCH is evident from Immunodeficiency, Centromeric instability and Facial anomalies (ICF). This disorder, which is characterized by decondensation and rearrangements of PCH regions, is commonly caused by inactivating mutations in the gene encoding DNA methyltransferase 3B (21,22). There is also mounting evidence from cancer that hypomethylation is implicated in destabilizing PCH (23–25). Finally, instability of PCH can be induced by inhibition of DNA methylation with DNA methyltransferase inhibitors (15). Thus, DNA methylation seems to be essential for preserving stability of heterochromatin satellite DNA.

We and others have shown that hypomethylation of 1q12 PCH in some premalignant and malignant cells promotes an epigenetic reprogramming by Polycomb-group (PcG) proteins (18,26). PcG proteins are chromatin repressive factors normally enriched on facultative heterochromatin together with H3K27me3 and H2AK119ub. There are at least two categories of PcG complexes, designated PcG repressive complex 1 and 2 (PRC1 and PRC2), which are found as different variants with distinct compositions and functions (27). PRC1 comprise four core components of the Cbx, Ring1, Phc, and Bmi1 type proteins, respectively. The complex can specifically recognize H3K27me3 catalyzed by PRC2 and has E3 ubiquitin ligase activity for H2A. The PRC2 complex contains the EZH2, EED and SUZ12 type of proteins and specifically interacts with H2AK119ub produced by PRC1. Initially, PRC2 deposition on chromatin was believed to exclusively prime PRC1 recruitment, but recent evidence suggests a more complex mechanism for PcG deposition. PcG factors are important players in repression of facultative heterochromatin in regulation of mammalian cell identity. In addition, PcG proteins also have a controversial role in regulation of PCH (28,29). Several studies have demonstrated that PcG proteins can bind pericentromeric satellite DNA, mainly in the absence of DNA methylation (30–34), and may be required for constitutive heterochromatin formation (32). In accordance with this, we have previously demonstrated that PRC1 factors accumulate on chromosome 1q12 PCH in response to demethylation of satellite DNA (35). These subnuclear domains, termed PcG bodies, may serve to maintain satellite DNA stability subsequent to loss of DNA methylation in cancer (16,19,36). Another possibility is that PcG bodies repress senescence, since unfolding of satellite heterochromatin has been proposed as a hallmark of senescent cells (37).

In a recent publication, we demonstrated that SSX2 interferes with the stability of PcG bodies (38). The SSX family comprises nine highly identical proteins with additional splicing variants, strictly expressed in the spermatogonia of testis in healthy individuals, but ectopically expressed in melanoma and many types of carcinoma and sarcoma (38–40). This cancer/testis-associated expression profile has made SSX proteins promising targets for cancer therapy (41). The cellular functions of members of the SSX family remain poorly understood, but we and others have demonstrated that SSX proteins support the growth of cancer cells (42–44) and have the ability to induce senescence, similar to oncogenes such as BRAF and KRAS (42). SSX proteins share two conserved domains, the Krüppel associated box (KRAB) and SSX repression domain (SSXRD), which repress reporter gene expression (45,46). The former is a classical protein-binding domain that interacts with SSX2IP and RAB3IP (47), while the latter seems to be involved in directing SSX proteins to PcG chromatin structures (48). The chromatin-association of SSX proteins may be established by direct association with DNA, since SSX2 possesses DNA binding activity *in vitro* (38). In synovial sarcoma, the SSXRD is spliced to SS18, a component of the SWI/SNF chromatin remodeling subcomplex GBAF, to form a fusion oncogene that is essential for the etiology of this disease. The oncogenic property of this fusion protein is at least partially based on its ability to hijack BAF complexes to PcG chromatin domains (49). In another study, SS18-SSX was demonstrated to bind the histone demethylase KDM2B, a subunit of a the non-canonical PRC1.1 complex, and thereby redirect SWI/SNF complexes to demethylated CpG islands, consequently causing deregulation of developmental programs to drive transformation (50).

The results presented herein implicate SSX proteins in destabilization of PcG-repressed 1q12 PCH leading to genomic instability and suggest a possible role for SSX proteins in cancer initiation and/or progression.

## MATERIALS AND METHODS

### Cell culture

A375, FM28 and MCF7 cells were grown in RPMI (Sigma Aldrich, Brondby, Denmark), 10% FBS and penicillin/streptomycin. The FM28 melanoma cell line was originally established by A. Kirkin and kindly donated by Professor MH Andersen, Center for Cancer Immunotherapy (CCIT), Herlev Hospital, Denmark. Cell lines were kept at low passage and cultured for no more than 3 months. When relevant, cell identities according to ATCC were verified using DNA fingerprinting by short tandem repeat (STR) analysis (Cell IDTM system. Promega). Cell lines were frequently tested for mycoplasma (MycoAlert, Mycoplasma detection kit, Lonza). For blocking replication or mitosis prior to SSX2 expression, cells were treated with 10 μg/ml of aphidicolin (Sigma Aldrich) or 100 mM nocodazole (Sigma Aldrich), respectively, for two hours before addition of 100 ng/ml doxycycline. Replication was measured by incorporation of EdU using the Click-iT EdU imaging kit according to the manufacturer's recommendations (Thermo Fisher Scientific, Hvidovre, Denmark). The activity of EZH2 was blocked by addition of 10 μM EPZ6438 (Selleckchem, Munich, Germany).

### Ectopic SSX2 expression

Generation and culture conditions for A375-SSX2 and MCF7-SSX2 cells with doxycycline-inducible SSX2 expression were described previously (38). For transduction of FM28 cells, SSX2 was inserted into the multicloning site of pCDH-CMV-Puro-GFP. The plasmid was packed in lentivirus by cotransfection with pMD2.G, pRSV-Rev, pMDL g/p RRE (kindly provided by the Trone Lab through Addgene, Cambridge, UK) into HEK293T cells using Lentifectin (Abmgood, Richmond, Canada). Virus was harvested from the supernatant after 3 days, filtered, precipitated with PEG and resuspended in PBS. FM28 cells were infected overnight and prepared for Immunostaining-fluorescence in situ hybridization as described below.

Expression of SSX1, SSX3, SSX4 and the alternative splice variant of SSX2 (please refer to Supplementary Figure S1 for sequences) in A375 cells was done with pLX304 cDNA expression plasmids obtained from the DNA resource core at Harvard Medical School, Boston, USA and transfection with Optifect according to the manufacturer's recommendations (Thermo Fisher Scientific).

Deletion or point SSX2 mutants were generated by PCR from a SSX2 ORF template. Point mutations were introduced by using mutated internal primers together with end primers to generate overlapping products, which were assembled in a second PCR. ORFs were either inserted into the pcDNA6.2-EmGFP plasmid for expression with N-terminal emGFP tag using TOPO cloning (Life Technologies) or pLVX-TetOne-puro plasmid (Takara, Mountain View, CA, USA) for docycycline-inducible expression using InFusion cloning. Plasmids were prepared as lentivirus as described above and used for infection of A375 cells. After transduction, stably transduced cells were selected with 0.6 μg/ml puromycin. Expression of SSX2 was induced by addition of 2 μg/ml doxycycline to the media.

### Fluorescence microscopy

Cells grown on coverslips were fixed in 4% formaldehyde and permeabilized in 0.2% Triton X100, PBS. For staining, cells were blocked in 3% BSA, PBS and immunostained with indicated antibodies, including anti-SSX2/SSX3 (clone 1A4; Sigma Aldrich; 1:100), rabbit anti-BMI1 (Cell Signaling, MA, USA; 1:200), mouse anti-BMI1 (ab14389; Abcam; 1:100), anti-histone H3K27me3 (Cell Signaling; 1:200), anti-histone H2AK119ub (Cell Signaling; 1:100), anti-RING1B (Cell Signaling; 1:100), mouse anti-CENP-B (C-10; Santa Cruz Biotechnology, Heidelberg, Germany; 1:100), mouse anti-FBZL10/KDM2B (Novus; clone 5G1; 1:100; cells fixed in 100% methanol), mouse anti-FLAG M2 (Sigma Aldrich;; 1:1000) and rabbit anti-V5 (Cell Signaling; 1:100) (51). Cells were then incubated with secondary antibodies: goat anti-mouse IgG (H+L) cross-adsorbed Alexa Fluor 488/568 (Thermo Fisher Scientific) and goat anti-rabbit IgG (H+L) cross-adsorbed Alexa Fluor 488/568 (Thermo Fisher Scientific) and mounted under cover slides with ProLong Gold Antifade with DAPI (Life Technologies). Imaging was performed with a PlanApo N 60×/1.42 oil objective fitted on an Olympus IX73 microscope. The specificity of primary antibodies is documented by the manufacturer and in our previous paper (38). Control experiments were performed initially to test for species specificity of secondary antibodies. At least 80 cells were scored in triplicates for each data point in phenotype assays. Line scans were performed with CellSens Entry software (Olympus).

### Immunostaining-fluorescence in situ hybridization

Cells were attached to microscope slides using cytospin (500 g for 5 min), fixed in 4% formaldehyde for 10 min and stained as above. After another fixation in formaldehyde, cells were incubated in 2× SSC (Sigma Aldrich), 0.05% Tween (pH 7) for 2 min and dehydrated for 2 min per step in a series of 70, 85 and 100% ethanol. Air-dried cells were heated with 1q12 satellite III FISH probe in hybridization buffer (Cytocell, Cambridge, UK) under a coverslip, sealed with rubber cement (Elmers, High Point, NC, USA), for 2 min at 75°C and incubated for 37°C for 16 h in a humidified atmosphere. Then cells were washed for 2 min in 0.25xSSC (pH 7) and two times 2 min with 2xSSC, 0.05% tween (pH 7). Mounting on glass slides was done with Prolong gold antifade mount with DAPI (Thermo Fisher Scientific). The length of 1q12 satellite III domains was measured using CellSens Entry software (Olympus) (*n* > 80).

For RNA-FISH, cells were stained with BMI1 antibodies as above (with the addition of 1u/μl RNAsin, Thermo Scientific to buffers), treated with 100% ethanol for 10 min and air dried. Cells were incubated with 50 pm/μl probe in hybridization buffer [4× SCC, 0.2% ultrapure BSA (Thermo Scientific), 20% dextran sulfate] at 37°C for 16 h in a humidified atmosphere. Cells were washed in 2× SSC, 15% formamide for 20 min at 37°C, then in 2× SSC for 20 min for 37°C, then in 1× SSC for 20 min at room temperature, and finally in 4× SSC for 5 min at room temperature. Mounting on glass slides was done with Prolong gold antifade mount with DAPI (Thermo Fisher Scientific).

### RING1A/B shRNA knockdown

pLKO1-puro shRNA constructs for knockdown of RING1A (#21989) and RING1B (#33697) (Sigma Aldrich) were prepared as lentivirus, as described above. A375 cells were co-infected with both constructs and selected with 0.6 μg/ml puromycin. Knockdown of RING1A and RING1B and PcG body-depletion was verified by immunostaining, as described above.

### Growth assays

Cells were seeded in 6 well plates (500 cells/well) with or without 2 μg/ml doxycycline. After 8–10 days, plates were washed in PBS and fixed in 0.5% crystal violet, 25% methanol solution for 10 min. After washing in H_2_O, plates were air dried and scanned. Quantification was done by solubilizing crystal violet stain in 3% citrate, ethanol buffer and measuring OD570.

### Satellite DNA transcription quantitative PCR

RNA was purified using RNAzol (VWR, Soborg, Denmark) and concentrations were determined using BCA assay (Thermo Fisher Scientific). For quantitative measurement of satellite expression, 2 μg of DNAse-treated RNA was used for cDNA synthesis with Maxima H Reverse transcriptase (Thermo Fisher Scientific). Five microliter of cDNA was used for qPCR analysis using SYBR green mastermix (Qiagen, Copenhagen, Denmark). PCR cycles were 40 cycles of 15 s at 94°C, 30 s at 60°C and 30 s at 72°C. The satellite II primers were specific for pericentromeric satellite II repeats on chromosome 1, 9 and 10. Satellite III primers specifically recognize satellite III repeats of the chromosome 1q12 region were adapted from Enukashvilly et al. (25). The SATIII amplicon is contained in the BAC clone RP11-327C24 (GenBank Accession No. AC079934.3). α-Satellite primers were specific for a centromeric satellite repeat on chromosome 1. Please refer to the supplementary material for primers.

Fold changes were calculated 2^(Ct – DOX-Ct + DOX)^. Due to the need for specific priming of cDNA reactions no reference gene was used for normalization. Instead input RNA levels were normalized based on NanoDrop spectrophotometer measurements. The quality of RNA was checked by a separate cDNA synthesis with 2 μg RNA and random primers and subsequent analysis of PUM1 expression. This analysis showed highly similar results for all samples. For all reactions, minus RT and no template controls were included.

### Western blotting

Extracts were made from sub-confluent monolayers of cells with RIPA buffer, resolved by 4–20% SDS-PAGE and electroblotted onto a PVDF membrane. The membrane was incubated in PBS, 0.1% Tween-20 and 5% non-fat dry milk powder to block remaining protein binding sites, and then incubated with anti-SSX2-4 (1:3000) (52) or anti-ZBED1 (Sigma Aldrich, wh0009189M1, 1:2000). The blot was further stained with horseradish peroxidase-conjugated goat anti-mouse IgG (DakoCytomation Denmark A/S, Glostrup, Denmark) and developed with ECL Western Blot kit (Amersham Biosciences, Hilleroed, Denmark, 1:100 000). All antibody incubation and washing steps were carried out in PBS, 0.1% Tween-20.

### Bisulfite sequencing

Analysis of DNA methylation was conducted by bisulfite sequencing as described previously (35). Primers for PCR amplification of bisulfite-treated satellite II DNA are provided in the supplementary material.

### SSX2 FRAP analysis

A375 cells were transfected with pcDNA6.2-EmGFP-SSX2 plasmid for expression of SSX2 with an N-terminal emGFP tag using Optifect according to the manufacturer's recommendations (Thermo Fisher Scientific). Cells were cultured in phenol red-free DMEM (Life Technologies), 10% FBS and penicillin/streptomycin for 16 h before analysis. Fluorescence recovery after bleaching (FRAP) analysis was performed using an Olympus BXG1Wl confocal microscopy with a LUMPLFL 60x NA:0.90 objective. After a pre-bleach scan, cells were bleached for 150 ms with the 488 nm laser and scans were collected every 10 s. Fluorescence intensities (ex 488 nm) were expressed relative to pre-bleach levels.

### ChIP-qPCR

Chromatin Immunoprecipication (ChIP) experiments were performed as previously described (53). Briefly, A375 cells with or without ectopic SSX2 expression were crosslinked in 1% formaldehyde and sonicated according to the manufacturer's protocol using the Covaris ME220 Focused-ultrasonicator. Equal amounts of sheared chromatin were incubated with 2 μg/μl BSA (Sigma, Aldrich), 1× complete protease inhibitors (Roche, Hvidovre, Denmark) and antibody at 4°C for 3 h, followed by overnight incubation with protein A beads at 4°C, rotating. The antibodies anti-BMI1 (6964; Cell Signaling, 1:50), anti-H3K27me3 (9733; Cell Signaling, 1:50), anti-H2AK119ub (8240; Cell Signaling, 1:100), anti-H3 (4620; Cell Signaling, 1:50), anti-H2A (12349; Cell Signaling, 1:100) and normal rabbit IgG (2729; Cell Signaling, 1:200) were used for immunoprecipitation. Immunoprecipitated DNA and 5% input DNA was analyzed by quantitative real-time PCR using SYBR green mastermix (Qiagen, Copenhagen, Denmark) and primers specific for the satellite II and satellite III regions (for primer sequences, see supplementary materials). The immunoprecipitated DNA was amplified for 40 cycles of 15 s at 95°C, 15 s at 60°C, and 15 s at 72°C and quantification of immunoprecipitated DNA was calculated as percentage of input DNA.

### Overexpression of satellite II transcripts

Satellite II PCR products, amplified from cDNA from A375-SSX2 cells as described above, were analyzed by gel electrophoresis and the major products (∼160 bp) were cloned into the pLenti7.3 expression plasmid (Life Technologies) under the control of a CMV promoter. Plasmids were prepared as lentiviral particles as described above and used for infection of A375 cells.

### LNA GapmeR transfection

A375-SSX2 cells were transfected with 25 nM each of LNA GapmeR using Optifect (Life Technologies) according to the manufacturer's recommendations. A mix of the GapmeRs targeting both the sense and antisense transcripts were used. As control, a non-targeting GapmeR was used in an equal concentration. Please refer to the supplementary materials for GapmeR sequences.

### Electromobility shift assays (EMSA)

The production of recombinant SSX2 for these experiments has been published previously (38). For generation of methylated and unmethylated versions of the same DNA fragment, a plasmid containing 75 base pairs of the internal control region from the somatic 5S rRNA gene from Xenopus Laevis inserted in the BamHI site of pUC 19 was used as template for PCR reactions. PCR products were obtained by using M13 reverse and the M13 forward primers labelled with ^32^P-g-ATP by polynucleotide kinase, 10 ng plasmid and 200 mM dGTP, dCTP (or 5-methyl dCTP), dATP and dTTP. This resulted in two fragments of 180 bp, one with normal nucleotides and one with 5-methyl dCTP. The fragments were purified on polyacrylamide gels, electroeluted from the gel, precipitated with ethanol and subsequently dissolved in the SSX2 binding buffer containing 20 mM Tris, pH 7.4, 50 mM NaCl and 1 mM DTT. The satellite II DNA fragment was synthesized and inserted into the EcoR1 and BamH1 sites of the pUC57 plasmid. After plasmid amplification, the fragment was released by EcoR1 and BamH1 digestion, gel purified and labelled with ^32^P. Please refer to the supplementary materials for the DNA fragment sequence.

For gel shift assays, DNA fragments were incubated with the indicated concentrations of SSX2 in the binding buffer, loading buffer was added and samples were immediately loaded on 5% polyacrylamide gels and run at 6–8 V/cm at 238°C for 90–120 min. Following electrophoresis, the SSX2-DNA complexes were detected by a phosphoimager.

### Northern blotting

Total RNA (50 μg) was resuspended in 10 μl formamide loading buffer and separated on a 1.5% formaldehyde agarose gel for 3 h and 15 min at 100 V. Alternatively, 10 μg of total RNA was separated on a 6% polyacrylamide gel for 2 h at 300 V. Separated RNA was capillarity blotted onto a Zeta-probe nylon membrane (Bio-Rad, Copenhagen, Denmark) in 50 mM NaOH overnight followed by UV cross-linking. Membranes were pre-hybridized in PerfectHyb™ Plus Hybridization Buffer (Sigma Aldrich) for 20 min at 42°C before probing with ^32^P-labeled DNA single-stranded probes overnight. DNA primers used as probes for 1q12 sense/antisense satellite II or satellite III and 5S are listed in the supplementary materials. Probed membranes were washed three times in 2× SCC and 0.1% SDS for 10 min at 42°C, and bands were visualized using a Typhoon Trio (GE Healthcare) and analyzed with IQTL 8.0 software (GE Healthcare).

### Chromatin association assay

A375-SSX2 cells were growth for 24 h with 2 μg/ml doxycycline and suspended in 10 mM HEPES (pH 7.9), 340 mM sucrose, 3 mM CaCl_2_, 0.1 mM EDTA, 5 mM valproic acid, PhosStop (Roche) and Complete protease inhibitors (Roche) for 10 min to lyse cells. Nuclei were isolated by centrifugation and resuspended either in SDS-PAGE sample buffer (nucleus fraction) or in 10 mM Tris–HCl (pH 7.4), 0.2 mM MgCl_2_, 1% Triton X-100, 5 mM valproic acid, PhosStop (Roche) and Complete protease inhibitors (Roche) for 15 min. Nuclear lysates were centrifuged to isolate the chromatin fraction and supernatant was mixed with SDS-PAGE sample buffer and saved as nucleoplasma fraction. The insoluble chromatin fraction was resuspended in SDS-PAGE sample buffer and sonicated to reduce viscosity.

### Structure modulation

Structural modulation of SSX2 (CAA60111.1) based on known structures was carried out with the Phyre2 web portal (54) and visualized using the open source PyMOL molecular graphics system.

### Crosslinking of recombinant SSX2

Recombinant SSX2 was diluted to indicated concentrations in 20 mM PBS (pH 7.4), 50 mM NaCl and glutaraldehyde was added to a final concentration of 0.1%. After 2 min at 37°C, glutaraldehyde was quenched by addition of glycine. Oligomerization was analyzed by Western blotting.

### Statistical testing

Statistical analysis was carried out using two-tailed students t-tests in Prism 6 software. Statistical significance is indicated by asterisks: *P < 0.05; **P < 0.001; ***P < 0.0001.

### Primers and probes

A list of primers and probes can be found in the supplementary materials.

## RESULTS

### SSX2 causes epigenetic deregulation and transcriptional derepression of 1q12 pericentromeric satellite DNA

We have previously shown that SSX proteins can target PcG bodies in a concentration-dependent manner, and that this interaction is unstable, ultimately leading to the depletion of PcG bodies from cells (38) (Supplementary Figures S2 and S3). Because we and others recently found that PcG bodies form when PRC1 proteins accumulate on the 1q12 PCH in response to demethylation of this domain in premalignant and malignant cells (26,35), we speculated that SSX2-mediated depletion of PcG bodies might perturb the epigenetic control of the 1q12 domain. To test this, we used A375 melanoma cells, which have prominent PcG bodies.

**Figure 1.**
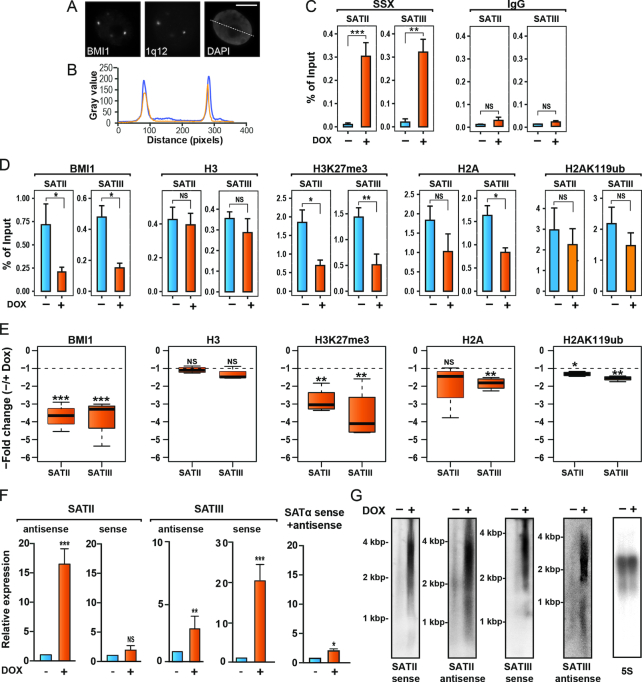
SSX2 promotes loss of PcG factors and transcriptional derepression of 1q12 pericentromeric satellite DNA. (**A**) Combined anti-BMI1 immunofluorescence and 1q12 satellite III probe FISH analysis of A375 melanoma cells demonstrates the presence of chromosome 1q12 pericentromeric heterochromatin domain in PcG bodies. (**B**) Line scan of A. Blue = BMI1, Yellow = SSX2. Scale bar = 5 μm. (**C**) SSX2 occupancy at 1q12 satellite II and III DNA in A375 cells was investigated by ChIP-qPCR analysis. Doxycycline (DOX)-induced SSX2 expression shows recruitment of SSX2 to investigated regions. As a negative control, IgG was used. (**D**) BMI1, histone H3, H3K27me3, histone H2A and H2AK119ub occupancy at 1q12 satellite II and III DNA in A375 cells. Induction of SSX2 expression in A375 cells by addition of doxycycline (DOX) reduced deposition of BMI1 and PcG-associated chromatin modifications at 1q12 satellite II and III repeat DNA, as demonstrated by ChIP-qPCR analysis. (**E**) Boxplots showing the fold changes between the occupation of factors and histone marks investigated in 1D with and without ectopic SSX2 expression are shown for individual experiments. (**F**) Doxycyclin (DOX)-induced SSX2 expression enhanced transcription of satellite II and III DNA, but not satellite-α DNA, as demonstrated by RT-qPCR analysis. (**G**) Northern blot demonstrates satellite II and III transcripts in A375 with SSX2 expression induced by DOX. Data represent the mean ± SD for three biological replicates (C-F). A two-sided t-test was used for statistical analysis (*P < 0.05, **P < 0.001, ***P < 0.0001).

First, we confirmed the deposition of PcG factors at 1q12 PCH in these cells. This megabase satellite domain contains multiple copies of satellite II and III repeats. Using a combined immunofluorescence-FISH staining, we found complete consistency in numbers and position between PcG bodies represented by the PRC1 protein BMI1 and the hybridization foci of a probe specific for satellite III repeats of 1q12 PCH (Figure 1A,B). A375 cells contained between 2 and 5 PcG bodies, consistent with their hypotriploid karyotype and karyo-instability. In normal cell types, the satellite III FISH probe recognize two chromosomal foci, confirming its specificity towards the PCH domain at 1q12 (18).

To gain insight into the role of SSX2 in the epigenetic control of 1q12 PCH, we first investigated the occupancy of SSX2, PRC1 (represented by BMI1) and PcG-associated histone modifications H3K27me3 and H2AK119ub at the 1q12 satellite repeats using ChIP-qPCR in A375 cells with inducible SSX2 expression. Importantly, ectopic SSX2 expression resulted in recruitment of SSX2 to the 1q12 II and III satellite repeats (Figure 1C). The PRC1 protein BMI1 was found to be associated with pericentromeric satellite III repeats at 1q12, and with pericentromeric satellite II structures found at chromosome 1, 9 and 16 (Figure 1D). BMI1 was not detected on α-satellite repeats of the chromosome 1 centromere (Supplementary Figure S4). BMI1 enrichment at these sites was accompanied by H3K27me3 and H2AK119ub, which generally exhibit a high correlation with PcG deposition (28). Thus, PcG factors indeed deposit on 1q12 heterochromatin satellite DNA, and possibly on chromosome 9 and 16 heterochromatin satellite DNA. We next investigated the effect of ectopic expression of SSX2 on the epigenetic landscape of 1q12 heterochromatin satellite DNA. Importantly, the recruitment of SSX2 resulted in a significant loss of PRC1 protein BMI1 and the polycomb-associated repressive histone mark H3K27me3 (Figure 1D and E). To determine if loss of H3K27me3 was a result of loss of histone H3, as seen during more local chromatin remodeling (such as enhancer activation), we profiled the occupancy of histone H3 in the 1q12 region with and without ectopic SSX expression. We found that histone H3 levels in this region were not affected by SSX2 expression, suggesting that SSX2 directly reduces H3K27me3 levels, either by antagonizing the deposition or by active removal of this repressive mark. Intriguingly, we also found that ectopic SSX2 expression resulted in a slight reduction in H2A and H2AK119ub deposition, although this small change may be below biological significance (Figure 1D and E).

Because of the repressive nature of PcG proteins, we hypothesized that SSX2-induced loss of PcG factors from 1q12 PCH would lead to transcriptional derepression of 1q12 satellite DNA. Thus, we investigated transcription levels from specific 1q12 satellite III repeats and chromosome 1, 9 and 16 pericentromeric satellite II DNA repeats, using the same primers as for ChIP-PCR. This demonstrated very low basal levels of satellite II and III sense and antisense transcripts in A375 melanoma cells with intact PcG bodies. Upon SSX2-mediated depletion of PcG bodies, a significant induction of satellite II sense and satellite III sense and antisense transcription was observed (Figure 1F). In contrast, no induction in transcription from chromosome 1 centromeric α-satellite was seen, in agreement with the BMI1-binding at this domain being epigenetically unaffected by SSX2 (Figure 1F). The transcription of 1q12 satellite II and III DNA was further investigated by northern Blotting, which revealed transcripts of a broad size-range of approximately 2.000-4.000 bp corresponding to both sense and antisense strands of satellite II and satellite III (Figure 1G). Satellite transcripts of different lengths have been identified in different studies, some similar to the size of the transcripts detected in SSX2-expressing A375 cells (55–59).

Taken together, these results suggest that SSX2 depletes PcG bodies from A375 cells by reducing PcG activity at 1q12 heterochromatin satellite DNA, thereby changing the epigenetic landscape to promote transcriptional derepression of 1q12 satellite DNA.

### SSX2 unfolds and destabilizes polycomb-repressed 1q12 PCH

**Figure 2.**
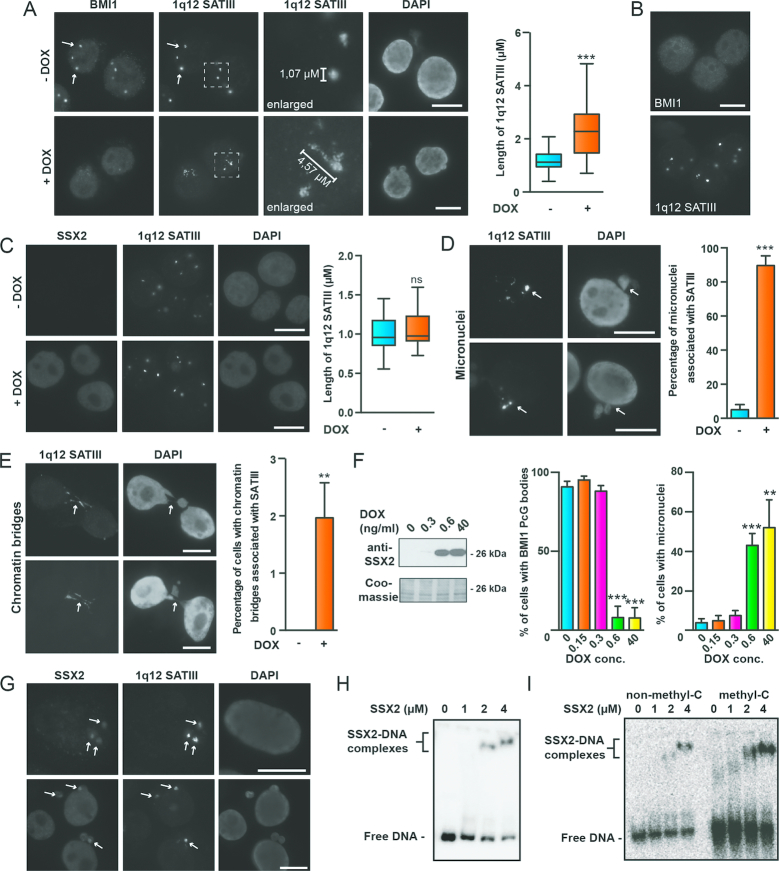
SSX2 unfold and destabilize 1q12 pericentromeric satellite DNA. (**A**) Combined immunofluorescence-FISH staining of BMI1 and 1q12 satellite III DNA demonstrates that induction of SSX2 expression in A375 cells by doxycycline (+DOX) leads to depletion of BMI1 PcG bodies and distention of 1q12 pericentromeric satellite DNA. Cells were stained 48 h after addition of doxycycline. Left, representative images. Right, box plot showing the average 1q12 satellite III length in >80 cells per condition. (**B**) Combined immunofluorescence-FISH staining of BMI1 and 1q12 satellite III DNA shows that MCF7 cells are devoid of BMI1 PcG bodies associated with the 1q12 pericentromeric heterochromatin domain. (**C**) SSX2 does not associate with nor change the structure of the 1q12 pericentomeric domain (satellite III probe) in MCF7 cells. SSX2 expression was induced by addition of doxycycline (+DOX) for 48 hours. Left, representative images. Right, box plot showing the average 1q12 satellite III length in >38 cells per sample. (**D**) 1q12 satellite III DNA is spatially associated with micronuclei (arrows) in A375 cells after 48 h of doxycycline-induced SSX2-expression. Left, representative images. Right, histogram showing the percentage of observed micronuclei associated with 1q12 satellite III in the cell population (>100 cells were quantified per sample). (**E**) 1q12 satellite III structures are found in chromatin bridges (arrows) in A375 cells after 48 hours of doxycycline-induced SSX2-expression. Data represent the mean ± SD for three biological replicates. Left, representative images. Right, histogram showing the percentage of chromatin bridges associated with 1q12 satellite III in the cell population (>100 cells were quantified per sample). (**F**) SSX2 exhibits concentration-dependent targeting and depletion of BMI1 PcG bodies in A375 cells that correlates with formation of micronuclei. Different levels of SSX2 expression were induced in A375 cells using different concentrations of doxycycline (DOX). After 48 hours, cells were stained for BMI1 and with DAPI to detect and quantify PcG bodies and micronuclei. Left, Western blot showing levels of SSX2 relative to DOX levels. Right, histogram showing microscopical quantification of percentage of cells (>100 per sample) with PcG bodies and micronuclei at different levels of DOX/SSX2 (details in Supplementary Figure S7). (**G**) SSX2 colocalized with 1q12 satellite III DNA in A375 cells before (top) and after (bottom) destabilization. (**H**) Electrophoretic mobility shift assay showing binding of recombinant SSX2 (38) to 10 pg of a 276-bp 1q12 satellite II DNA fragment (^32^P labeled). (**I**) Electrophoretic mobility shift assay showing binding of recombinant SSX2 to 10 pg of a GC-rich 180-bp pUC19 fragment (^32^P labelled). The fragment was generated by PCR with either cytosine or methyl-cytosine. A two-sided *t*-test was used for statistical analysis (*P < 0.05, **P < 0.001, ***P < 0.0001). Scale bars = 10 μm.

We then assessed whether 1q12 PCH structure was modified by SSX2. Indeed, we found that SSX2 expression in A375 cells prominently distended 1q12 PCH, transforming the round 1q12 satellite III foci into larger, irregularly shaped, structures (Figure 2A). A similar pattern was seen in other melanoma cells with PcG bodies (i.e. the FM28 melanoma cell line) (Supplementary Figures S5). This distension of the 1q12 PCH domain clearly occurred in cells that lost PcG bodies. We also investigated the potential effect of SSX2 on the structure of centromeres, but no change was observed (Supplementary Figure S6), suggesting a specific effect on pericentromeric satellite DNA. To determine whether the 1q12 PCH structure was modified by SSX2 only in the context of a PcG body, we ectopically expressed SSX2 in MCF7 cells, which lack PcG bodies (Figure 2B and C). Interestingly, we found that SSX2 did not associate with 1q12 satellite III DNA in these cells (Figure 2C). In agreement with this, there was no change in the structure of 1q12 satellite III foci upon SSX2 expression.

We recently found that expression of SSX2 in A375 melanoma cells induced genomic instability in the form of micronuclei and chromatin bridges by an unidentified mechanism (42). A closer inspection of cells with genomic instability revealed that almost every micronucleus in these cells was spatially associated with 1q12 satellite DNA (Figure 2D). 1q12 satellite domains were located either within the micronuclei or in the interface between the micronuclei and the main nuclei. This was in contrast to A375 cells without SSX2 in which micronuclei were found to a much lesser extent, and rarely associated with 1q12 satellite DNA. In A375 cells without SSX2, chromatin bridges were not observed, whereas approximately 2% of SSX2-expressing cells had chromatin bridges that were always associated with 1q12 satellite DNA (Figure 2E). Titration of SSX2 expression in A375 cells further showed a correlation between levels of SSX2 required for PcG body depletion and levels required for inducing genomic instability (Figure 2F and Supplementary Figure S7).

Collectively, this strongly suggests that derepression and unfolding of PcG-repressed 1q12 PCH is directly implicated in the genomic instability arising from SSX2 expression. This is in line with studies demonstrating that decondensation of PCH regions, induced by demethylation, can affect kinetochore orientation and attachment to the mitotic spindle, which, in turn, leads to formation of micronuclei spatially associated with the affected satellites (15,60). The direct role of SSX2 in the genomic instability arising from its expression was further highlighted by its constitutive presence on 1q12 heterochromatin during it destabilization (Figure 2G).

We have recently demonstrated that PcG bodies are established upon demethylation of 1q12 satellite DNA (18). This was supported by bisulfite sequencing of A375 and MCF7 1q12 satellite III DNA showing that A375 cells are significantly less CpG methylated at this site compared to MCF7 cells (Supplementary Figure S8). This suggests that the association of SSX proteins with the 1q12 PCH domain depends on the presence of PcG factors or associated patterns of chromatin/DNA modification. We previously demonstrated that SSX proteins have DNA-binding capacity (38), suggesting that SSX might directly bind to satellite DNA. Indeed, EMSA showed that SSX2 binds a 276-bp satellite II sequence, which is repeated multiple times in the 1q12 region (Figure 2H). We then tested the effect of cytosine methylation on SSX2 DNA binding. However, the binding of SSX2 to methylated and naked versions a CG-rich DNA fragment were similar (Figure 2I). Thus, SSX2 binds satellite DNA *in vitro* in a methylation-independent manner.

### SSX proteins contain a zinc finger-like domain essential for 1q12 PCH association and destabilization

**Figure 3.**
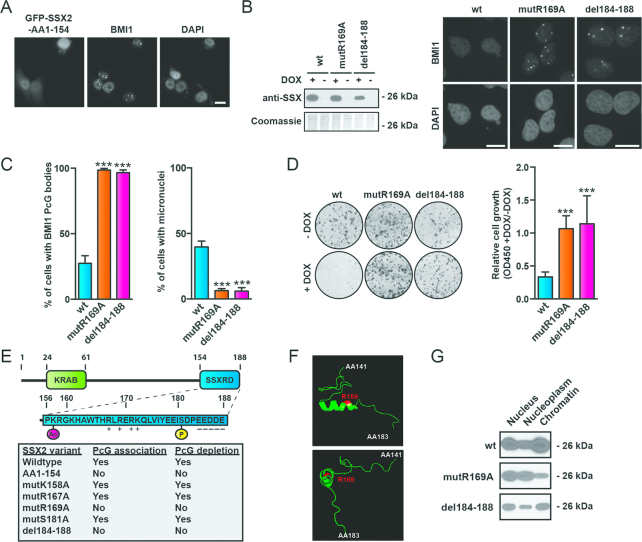
The SSXRD is required for both targeting of PcG bodies and destabilization of 1q12 PCH. (**A**) A SSX2 deletion mutant lacking the SSX2RD expressed with N-terminal GFP-tag (GFP-SSX2-AA1-154) did not associate with BMI1 PcG bodies. (**B**) Mutation of amino acid 169 (R169A) or deletion of the c-terminal amino acids 185–188 (del-184–188) of SSX2 prevents association and depletion of PcG bodies and induction of genomic instability as determined by micronuclei formation. Left, Western blot showing expression of wild-type or mutant SSX2. Right, representative pictures of A375 cells expressing SXX2 mutants. (**C**) Histogram showing quantification of the percentage of cells with PcG bodies and micronuclei from (B). (**D**) Mutation of amino acid 169 (R169A) or deletion of the c-terminal amino acids 185–188 (del-184–188) of SSX2 reconstitutes clonogenic growth of A375 cells with SSX2 expression. Expression of SSX2 mutants in A375 cells was induced by addition of doxycycline (+DOX) for 72 h before analysis of cell numbers by crystal violet staining. Left, representative wells with cells stained with crystal violet. Right, quantification of crystal violet staining. (**E**) Overview of SSX2 domains and amino acids identified as important for targeting and depletion of PcG bodies (details in Supplementary Figure S9). (**F**) The SSXRD contains an alpha-helical structure similar to the zinc finger C2H2 domain. This alpha helix contains arginine at position 169, which is essential for the association of SSX with PcG bodies. Structure modulation analysis was done with Phyre2 and visualized with PyMOL (see Supplementary Figure S10 for details). The structure is shown from different views. (**G**) Investigation of the chromatin association of SSX2 mutants where arginine-169 was substituted with alanine (R169A) or the negatively-charged C-terminal was deleted (del184–188). Only SSX2-R169A exhibited reduced chromatin binding. Chromatin was extracted from A375 cells and the level of copurified SSX was analyzed by Western blotting. A two-sided t-test was used for statistical analysis (*P < 0.05, **P < 0.001, ***P < 0.0001). More than 100 cells were analyzed per sample. Scale bars = 10 μm.

We next focused on identifying the mechanism by which SSX2 destabilizes 1q12 PCH. First, we investigated the structural basis for SSX2 targeting and depletion of PcG bodies. The ability of SSX proteins to target PcG bodies was delineated to the 32 amino acids present at the C-terminal, which equaled the SSX-repression domain (SSX-RD) (Figure 3A and E). The SSX-RD is highly conserved among SSX family members (90–100% identity) (Supplementary Figure S1) and retained in the SS18-SSX fusion oncogene (61), and was earlier assigned a repressive effect on gene transcription in a reporter assay (46), which may be explained by its association with PcG factors. To further characterize the structural basis for SSX2 targeting of PcG bodies, we scrutinized the C-terminal (AA155–188) for structural motifs with potential importance for molecular interactions (Supplementary Figure S9). Amino acids 167–172 contain a stretch of positively- charged amino acids, which could be implicated in protein-protein or protein-DNA interactions. Indeed, mutation of this site abolished the ability of SSX2 to target PcG bodies, and further analysis assigned this effect to the arginine at position 169 (Figure 3B and Supplementary Figure S9). In addition, deletion of five negatively-charged amino acids located in the very C-terminal end of the protein (del184–188) also perturbed PcG targeting (Figure 3B and Supplementary Figure S9). Mutation of acetylation site (K158A) and a phosphorylation site (S181A) did not affect this function of SSX2. Interestingly, mutants unable to associate with PcG bodies (R169A and del184–188) also did not produce genomic instability in cells (Figure 3C and D).

We have demonstrated that SSX2 have DNA binding capacity *in vitro*, but the domain responsible for this interaction has yet to be identified. Since the SSXRD seemed essential for SSX association with 1q12 PCH (Figure 3E), we speculated that this domain might be responsible for the interaction of SSX with satellite DNA. Interestingly, *in silico* structural modulation revealed a high probability that the fold of SSXRD resembles that of the classical C2H2 binding zinc finger domain, as the SSXRD was modelled to the C2H2 domains of several zinc finger domain proteins with high confidence (Supplementary Figure S10). The C2H2 domain is one of the most common DNA binding domains found in eukaryotic transcription factors. C2H2 zinc finger motifs (C2H2-ZNF) recognize DNA sequences by binding to the major groove of DNA via a short alpha-helix (70), and a similar short alpha-helix is present in the SSXRD (Figure 3F). Importantly, lysine-169 of the SSXRD, which was demonstrated to be essential for SSX2 association with 1q12 PCH/PcG bodies (Supplementary Figure S9), is located in this alpha helix (Figure 3F). Mutation of lysine-169 was further demonstrated to reduce SSX2 chromatin association in general (Figure 3G). Collectively, these data suggest that the SSXRD contains a zinc finger-like domain mediating binding of SSX proteins to DNA.

### The SSX KRAB domain is essential for SSX-mediated destabilization of 1q12 PCH

**Figure 4.**
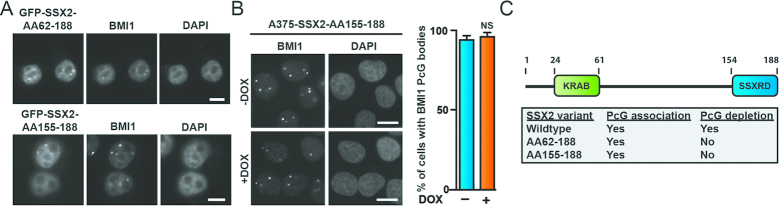
The SSX KRAB domain is required for depletion of PcG bodies. (**A**) SSX2 deletion mutants lacking the KRAB domain expressed with N-terminal GFP-tag (GFP-SSX2-AA62-188 and GFP-SSX2-AA155-188) associated with BMI1 PcG bodies. (**B**) Induction of SSX2-AA155-188 expression in A375 cells by addition of doxycycline (DOX) did not deplete PcG bodies after 48 h. **(C**) Overview of SSX2 domains identified as important for targeting and depletion of PcG bodies. A two-sided *t*-test was used for statistical analysis (*P < 0.05, **P < 0.001, ***P < 0.0001). More than 100 cells were analyzed per sample. Scale bars = 10 μm.

In addition to the C2H2 zinc finger-like SSX2RD domain all SSX proteins contain a conserved structure resembling a KRAB domain. This makes SSX proteins structurally similar to the ∼400 known KRAB zinc finger transcription factors (71). However, the SSX KRAB domain only expresses low similarity with canonical KRAB domains and do not interact with KAP1 (72). Thus, while the structure and the overall functionality of SSX proteins may resemble KRAB zinc finger transcription factors, the specific functions may be very different. We investigated the role of the SSX KRAB domain in 1q12 PCH association and destabilization. Interestingly, an SSX2 deletion mutants lacking the N-terminal, including the KRAB domain (SSX2-AA62–188 and SSX2-AA155–188), retained the ability to associate with PcG bodies, but did not destabilize 1q12 PCH (Figure 4A–C). This suggests that SSX proteins interact with 1q12 PCH through their SSXRD domain and rearrange the structure of 1q12 PCH via the KRAB domain. KRAB domains are classical protein-interaction structures and often recruit chromatin modifiers, most commonly the transcriptional repressor KAP1, to rearrange chromatin. However, the low identity of the SSX KRAB to canonical KRAB domains and the lack of KAP1 interaction suggests a different function mediated through different interactions. From previously published studies identifying interaction partners for SSX and SS18-SSX (www.thebiogrid.org), we identified a number of proteins that could potentially co-function with SSX in rearranging 1q12 PCH (including ZBED1, KDM2B and SSX2IP). ZBED1 was the only protein observed to accumulate in PcG bodies (Supplementary Figure S11A). However, knockdown of ZBED1 prevented neither the association between SSX2 and PcG bodies nor the development of SSX2-mediated genomic instability (Supplementary Figure S11B). SSX2 was previously demonstrated to interact with SSX2IP. Since this protein structurally resembles Structural Maintenance of Chromosomes (SMC) proteins, which are important factors in higher order chromosome, we also investigated the possible colocalization of this protein with PcG bodies (Supplementary Figure S11C). However, SSX2IP was largely restricted to the cytoplasm in A375 cells and showed no overlap with PcG bodies. Since KDM2B was recently demonstrated to recruit SS18-SSX to PcG domains, we also tested the subcellular localization of this protein (Supplementary Figure S11D). Again, this protein did not accumulate in PcG bodies, with or without SSX2 expression. Thus, neither of the identified SSX interaction partners with possible chromatin-associated functions seemed to be involved in the SSX-mediated destabilization of 1q12 PCH, and potential SSX co-factors remain unidentified.

Interestingly, SSX2 has been demonstrated to bind SSX2, SSX3, SSX5, SSX6 and SSX7, and interaction between other SSX molecules has also been documented (73), suggesting that SSX proteins form oligomers. Such complexes may contain several chromatin binding domains and mediate chromatin-chromatin interactions and regulation of chromatin architecture. Further evidence for SSX oligomerization came from crosslinking experiments with recombinant, purified SSX2, which suggested the existence of dimers and possibly higher oligomers (Supplementary Figure S12). Since the KRAB domain was shown to be essential for SSX2-mediated depletion of PcG bodies and unfolding of 1q12 PCH, we tested whether SSX2 forms multimers through the KRAB domains that may lead to restructuring of 1q12 PCH/PcG bodies. However, an SSX deletion mutant lacking the SSXRD, but retaining the KRAB domain, could not be recruited to PcG bodies by full-length SSX2 (Supplementary Figure S13), suggesting that SSX molecules do not interact though their KRAB domains at 1q12 PCH.

### SSX2-induced instability of the 1q12 PCH domain is not mediated by loss of PcG factors

**Figure 5.**
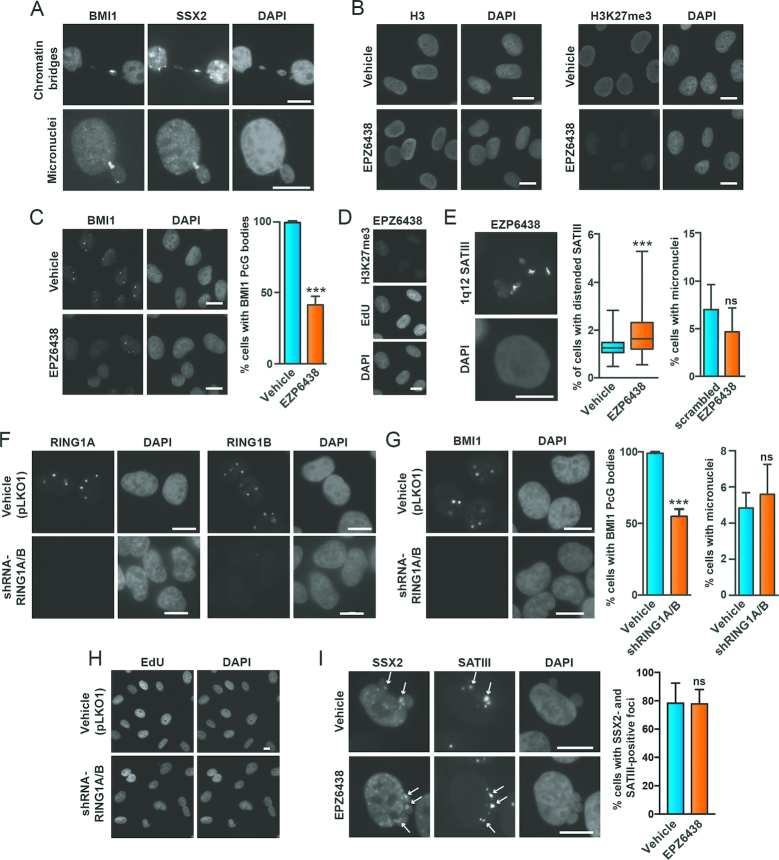
SSX2-mediated destabilization of 1q12 PCH is independent of PRC1 activity. (**A**) BMI1 is present together with SSX2 in chromatin bridges and micronuclei of A375 cells. (**B–D**) Inhibition of EZH3 with EPZ6438 reduces H3K27me3 (B) and depletes BMI1 PcG bodies (C), an effect not due to loss of cell proliferation (D). (**E**) Depletion of PcG bodies in A375 cells with the EZH2 inhibitor (EPZ6438 treatment for 96 hours) distends 1q12 satellite III DNA but does not induce genomic instability (i.e. micronuclei formation). (**F–G**) shRNA-mediated knockdown of RING1A/B in A375 cells (F) depletes BMI1 PcG bodies, but does not induce genomic instability (i.e. micronuclei formation) (G). (**H**) The effect in (F-G) is not due to loss of cell proliferation. (**I**) Depletion of PcG bodies in A375 cells does not prevent association of SSX2 with 1q12 satellite III or SSX2-mediated genomic instability. Data represent the mean ± SD for three biological replicates. A two-sided *t*-test was used for statistical analysis (*P < 0.05, **P < 0.001, ***P < 0.0001). More than 100 cells were analyzed per sample. Scale bars = 10 μm.

Although the above results suggested a causal relationship between loss of PcG repression and SSX2-mediated 1q12 PCH instability, this was contradicted by finding that PRC1 protein BMI1, in many cases, was maintained together with SSX2 at destabilized 1q12 satellite DNA (Figure 5A). We reasoned that if SSX2-induced unfolding and destabilization of 1q12 PCH is mediated by loss of PcG factors, then depletion of PcG bodies from A375 cells should phenocopy SSX2 expression. Therefore, we inhibited the H3K27me3 catalytic activity of EZH2, which is important for deposition of PRC1 and PRC2, and thereby depleted PcG bodies from A375 cells (Figure 5B-D), resulting in a deformation and size increase of 1q12 satellite III foci in many cells (Figure 5E). Despite this, no increase in the number of cells with micronuclei was observed. We also knocked down expression of RING1A and RING1B (Figure 5F and G), which are essential for the H2A-K119 ubiquitin E3 ligase activities of PRC1 and the deposition of PRC1 on chromatin (62), since we expected that this would deplete PcG bodies from cells. Silencing of RING1A/B indeed resulted in the depletion of PcG bodies from A375 cells (Figure 5H), but this did also not produce more cells with micronuclei. Next, we again depleted PcG bodies in A375 cells by inhibiting EZH2 and investigated if SSX2 associated with 1q12 satellite III DNA. We found that the frequency of cells with colocalization of SSX2 and satellite III was not affected by PcG depletion (Figure 5I). These results indicate that neither H3K27me3 nor PRC1/PRC2 deposition is essential for the stability of the 1q12 pericentromeric satellite DNA, and that depletion of PcG factors from 1q12 PCH is not implicated in the mechanism by which SSX2 causes its instability. Thus, PcG bodies seem to play a meric steric role in the observed phenotype. It further shows that PcG factors are not needed for SSX binding to this domain.

### SSX2-induced instability of the 1q12 PCH domain is not mediated by satellite DNA transcripts

Satellite derepression and transcription has been reported to enhance genomic instability in cancer cells. For instance, satellite II transcripts were implicated in satellite II repeat amplification (63), and a recent study showed that derepression of satellite DNA due to BRCA1 deficiency could induce genomic instability (64). Furthermore, JARID1C has been reported to contribute to heterochromatin maintenance and its loss induced expression of satellites that triggered genomic instability (65). We investigated whether 1q12 pericentromeric satellite transcripts mediated the genomic instability caused by SSX2. We knocked down satellite II and III expression using transfection with GapmeR pools targeting both sense and antisense transcripts (Supplementary Figures S14A and S14B). Satellite II knockdown did not affect PcG body stability or the ability of SSX2 to mediate genomic instability (Supplementary Figure S14C and D). In contrast, satellite III knockdown drastically reduced cell viability in the absence of SSX2 expression (Supplementary Figure S14B), but did not perturb SSX2-induced genomic instability in the surviving cells. We also expressed sense and antisense of a minor 160-bp satellite II fragment in A375 cells. Cells were cultured for 72 hours to allow for a replication-dependent effect on genomic stability. However, satellite II DNA over-expression did not affect the presence of PcG bodies or promote micronuclei formation in these cells (Supplementary Figure S14F and G). Although we also demonstrated that satellite II transcripts are associated with PcG bodies (Supplementary Figure S14H), the above results suggest that the genomic instability induced by SSX2 is independent of 1q12 satellite II and III transcription, and further supports a direct effect of SSX proteins on the structure and stabilization of 1q12 PCH. This conclusion is limited by the structural complexity of satellite repeats, leaving the possibilities that other 1q12 satellite transcripts (not recognized by the satellite II primers or GapmeRs used in this study) mediate the observed effects. Why reduced levels of satellite III transcripts lead to diminished survival of A375 cells remains elusive, but the cells showed no signs of PcG body destabilization or genomic instability (i.e. micronuclei), suggesting that the mechanism is unrelated to the stability of 1q12 satellite DNA (Supplementary Figure S14C).

### SSX2 exhibits a replication-dependent depletion of PcG bodies and destabilizes 1q12 pericentromeric satellite DNA in mitosis

**Figure 6.**
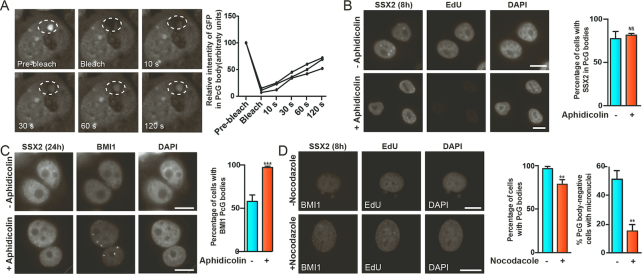
SSX2-mediated reorganization of 1q12 PCH/PcG bodies is replication-dependent. (**A**) FRAP analysis of the interaction of GFP-SSX2 with PcG bodies in A375 melanoma cells demonstrates a dynamic interaction between SSX2 and 1q12 PCH/PcG bodies. After bleaching the area containing PcG bodies (large dashed circle), reappearance of the fluorescence signal in the area corresponding to the PcG body was measured (small dashed circle). Quantification of the relative intensity of GFP in three arbitrary selected PcG bodies are shown. (**B**) Aphidicolin-mediated blocking of replication in A375 cells with SSX2 expression does not prevent targeting of 1q12 PCH/PcG bodies by SSX2. Aphidicolin was added to cells two hours before addition of docycycline (+DOX) for another 8 h for induction of SSX2 expression. Complete blockage of replications was shown by lack of EdU incorporation. Aphidicolin and EdU were added 2 h before induction of SSX2 with doxycycline. (**C**) Aphidicolin-mediated blocking of replication in A375 cells with SSX2 expression prevents depletion of BMI1 PcG bodies. (**D**) Nocodazole-mediated blocking of mitosis in A375 cells with SSX2 expression does not prevent depletion of BMI1 PcG bodies, but reduces genomic instability (i.e. micronuclei) in PcG body-negative cells. Data represent the mean ± SD for three biological replicates. A two-sided *t*-test was used for statistical analysis (*P < 0.05, **P < 0.001, ***P < 0.0001). More than 100 cells were analyzed per sample (B–D). Scale bars = 10 μm.

Looking further into the dynamics of SSX interaction with 1q12 PCH (visualized by PcG body staining), we found that SSX2 associated with these structures within hours after induction of SSX2 gene expression (Supplementary Figure S15). On the other hand, PcG bodies was not depleted in most cells until 24–48 hours after induction of SSX2 expression (Supplementary Figure S2), suggesting a mechanistic uncoupling of these events. To gain insight into how SSX proteins structurally change and derepress 1q12 PCH, we first performed FRAP analysis of SSX2 interaction with 1q12 PCH/PcG bodies. N-terminally emGFP-tagged SSX2 was expressed in A375 cells and cells with co-localization of SSX2 and 1q12 PCH/PcG bodies were identified. After photobleaching of 1q12 PCH/PcG bodies, SSX2 recovery was measured, demonstrating that SSX2 exhibits a rather fast exchange with 1q12 PCH/PcG bodies, replacing most proteins within minutes (Figure 6A). To explain the delayed depletion of PcG bodies upon SSX2 expression, we assessed whether replication was important for this effect. As expected from the results of the FRAP analysis, blocking replication with aphidicolin did not prevent SSX2 targeting of 1q12 PCH/PcG bodies (Figure 6B). It did, however, prevent the depletion of PcG bodies from cells (Figure 6C), strongly suggesting that replication is required for SSX2-induced structural reorganization of 1q12 PCH. Since aphidicolin-mediated blocking of replication would also prevent most cells from undergoing mitosis, we investigated the effect of blocking mitosis with nocodazole on the presence of PcG bodies and stability of 1q12 PCH. Importantly, although the blocking of mitosis did not prevent PcG body depletion, it significantly prevented formation of micronuclei (Figure 6D). This strongly suggests that 1q12 PCH is destabilized by SSX2 during replication and that this is associated with defective segregation of 1q12 PCH during anaphase, engulfing these structures in micronuclei. This is in accordance with earlier reports implicating hypomethylated 1q12 satellite DNA in chromosomal missegregation during anaphase and subsequent micronuclei formation (21,66–69).

## DISCUSSION

**Figure 7.**
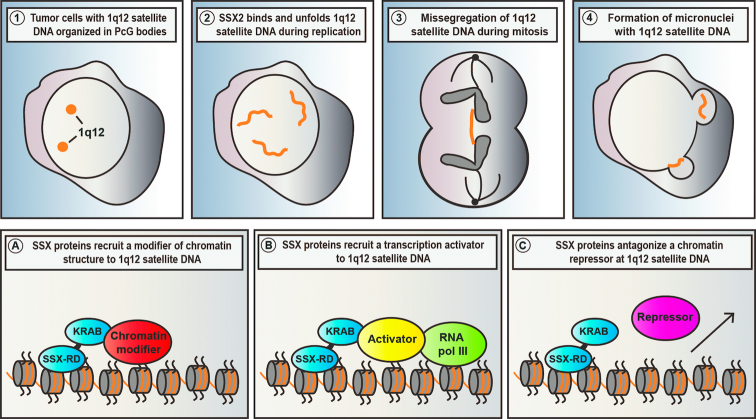
Proposed model of SSX-induced destabilization of 1q12 PCH. (Top panels) Our results demonstrate that SSX proteins targets 1q12 satellite DNA in the context of PcG bodies in tumors cells (1) and unfolds these structures during the replication phase (2). This leads to destabilization of 1q12 satellite DNA observed in the form of chromatin bridges during mitosis (3) and subsequent formation of micronuclei (4). (Bottom panels) There are different mechanism whereby SSX protein may destabilize 1q12 PCH. SSX proteins may recruit chromatin modifiers that facilitate chromatin reorganization, causing the unfolding and destabilization of this domain (**A**). SSX proteins may indirectly unfold and destabilize 1q12 PCH by activating satellite DNA transcription, either by anchoring transcriptional activators (**B**) or antagonizing repressors (different from PcG proteins) (**C**).

Genomic stability is compromised in most types of cancer and may support cancer development and progression. To expose vulnerabilities and identify novel therapeutic target points, it is important to provide insight into the mechanisms of genome deregulation in cancer cells. Instability of 1q12 PCH is a common phenomenon in cancer that may predispose to cancer-promoting chromosomal rearrangements. The cellular traits arising from such rearrangements and the resulting gain of 1q material remains elusive. However, the clonal evolution of cells with such aberrations suggest a role in supporting cancer phenotypes (3,5,74). In this study, we reveal a novel mechanism whereby ectopic expression of germ cell SSX proteins, as occurs in many different types of cancers, can structurally modify and perturb the stability of 1q12 PCH. We and others have recently demonstrated that 1q12 PCH is epigenetically reprogrammed into a PcG domain (i.e. PcG body) in premalignant and malignant cells (26,35). The results of the present study further demonstrate that SSX proteins can perturb the organization of PcG body-associated 1q12 PCH with genomic instability as an endpoint. Our results suggest a mechanism wherein SSX proteins bind chromatin of PcG bodies and, upon DNA replication, restructure this domain, leading to loss of PcG factors and chromosome destabilization (summarized in Figure 7 top panels). This provides a novel mechanism for promoting fragility of 1q12 PCH in the context of deregulated chromatin of cancer cells that may improve our understanding of how 1q duplications and translocations arise.

Our study reveals important characteristics about the chromatin-binding of SSX proteins. We have demonstrated that SSX2 binds DNA *in vitro*, including 1q12 satellite DNA, and we have found indications for a zinc finger-like structure in the SSXRD that may mediate this binding. This strongly suggest that SSX protein interact with chromatin through a direct binding to DNA. Our results from this study and previously published results (18) further suggest that SSX proteins exhibit at rather unselective binding to DNA *in vitro*. This raises the question of how the chromatin/DNA binding-specificity of SSX molecules is regulated in cells. SSX seems to strictly associate with 1q12 satellite DNA when this domain is structurally embedded in PcG bodies and characterized by DNA demethylation and deposition of PcG-associated histone marks and PcG factors. Surprisingly, DNA methylation alone did not interfere with SSX2 DNA binding *in vitro* and inhibition of suppression of PcG activity by inhibition of EZH2 and RING1A/B did not abrogate SSX2 binding to 1q12 satellite DNA (Figure 5H). Instead, the association of SSX with 1q12 satellite DNA *in vivo* may depend on the overall accessibility of these chromatin domains or be regulated by non-PcG factors.

The exact mechanism by which SSX proteins reorganize chromatin remains controversial. Our study demonstrates that SSX2 restructures 1q12 PCH during S-phase. This could be achieved by interfering with epigenetic memory during DNA replication, for instance by perturbation of the functions of enzymes or chaperones important for establishment of *de novo* histone modifications. While this may indeed be true, our results suggest that PcG factors are not lost during replication in the presence of SSX2, but may rather be lost as a subsequent event and therefore PcG bodies play a merely steric role in the development of the observed phenotype. This was supported by results showing that inhibition of PcG activity and loss of associated histone modifications could not phenocopy SSX2. Instead, SSX proteins may act on other factors that regulate 1q12 PCH structure or transcriptional activity. We have identified the SSX KRAB domain as essential for the SSX-mediated reorganization of 1q12 PCH. Interestingly, this domain has been shown to promote transcriptional repression (46), which seems to contradict our finding that this domain is important for derepression of 1q12 PCH. However, as for other proteins that regulate chromatin, the net functions may be context-dependent. The KRAB domain contained in SSX proteins exhibit only low similarity to canonical KRAB domains, and the molecular interactions they mediate also seem different, as SSX does not bind the classical KRAB-interacting protein and chromatin repressor KAP1. Despite our efforts to isolate SSX interactions partners that cooperate with SSX to establish derepression and unfolding of 1q12 PCH, such cofactors remain unidentified. However, we speculate that SSX proteins act by directly recruiting chromatin modifiers to affect chromatin structure or inducing satellite DNA transcription by stabilizing transcriptional activators or destabilizing transcriptional deactivator (Figure 7).

In addition to the possible mechanisms of SSX-mediated reorganization of 1q12 PCH noted above, there is also the option that SSX proteins act as non-histone, architectural molecules as, for instance, is seen with high mobility group proteins (HMG) (75). As demonstrated, SSX does not replace histones, but may still affect the structure of DNA to cause reorganization of chromatin. We have previously demonstrated that SSX2 exhibits a strong association with chromatin similar to, for instance, histones (38). Furthermore, SSX2 forms oligomers, at least *in vitro*, suggesting that every structural SSX unit may contain several DNA binding domains that may promote the ability to affect chromatin architecture.

Two recent studies have implicated satellite transcripts in generation of genomic instability. JARID1C was found to be functionally implicated in maintenance of heterochromatin, and its loss promoted genomic instability mediated by satellite transcripts (76). Similarly, BRCA1 loss was shown to compromise heterochromatin structure, leading to satellite DNA transcript-mediated genomic instability (64). We found that SSX-induced unfolding of 1q12 satellite DNA was associated with increased transcription from satellite II and III repeats. To test the importance of this, we knocked down 1q12 satellite expression during SSX-induced genomic instability, which did not diminish the observed phenotype. We also overexpressed a fragment cloned from 1q12 satellite II transcripts in cells without SSX2 expression with no effects on PcG body stability. This suggests that satellite transcripts are not important in SSX-induced genomic instability. Studies with JARID1C and BRCA1 were performed in mouse or primary human cells, whereas our data was generated with A375 melanoma cells. The latter constitutively express low levels of satellite transcripts and may have adapted to this state, possibly accounting for the discrepancy. In accordance with this, A375 cells exhibited greatly reduced viability in response to knockdown of 1q12 satellite III expression, but with no apparent effects on PcG bodies. Although, our results suggest that satellite transcripts are not implicated in the SSX2-mediated destabilization of 1q12 PCH, it is possible that the process of satellite transcription in itself may be involved.

In conclusion, our results demonstrate a previously unappreciated role for SSX proteins in enhancing genomic instability in cancer cells with PcG bodies. SSX genes are ectopically expressed in multiple cancer types due to promoter hypomethylation and are, as many other cancer/testis antigens, associated with disease progression (41). Interestingly, the described effects of SSX proteins on genome stability seem to be cancer-specific, as PcG bodies are not present in germ cells, which is the only SSX-positive normal type of cell. The results of this study, and earlier results showing that SSX proteins support cancer cell proliferation (42,43), suggest that SSX proteins may support cancer development and progression. This highlights SSX proteins as highly specific therapeutic cancer targets for targeted immunotherapy or small-drug inhibitors. Our results further provide mechanistic insight into regulation and deregulation of 1q12 PCH and emphasize the importance of satellite repeat domains as sites of chromosomal instability.

## Supplementary Material

gkz396_Supplemental_Files
