# Supplementary material for: Remodeling and destabilization of chromosome 1 pericentromeric heterochromatin by SSX proteins
Source: Nucleic Acids Res. 2019 May 22;47(13):6668–84. doi: 10.1093/nar/gkz396 (PMC6648343; doi:10.1093/nar/gkz396)
Supplement: gkz396_Supplemental_Files [file gkz396_supplemental_files.zip › Oligos.docx]

**SSX2 CLONING PRIMERS**

**Primers for cloning SSX2 mutants into the pcDNA6.2-EmGFP plasmid**

| SSX2 mutant | Forward primer | Reverse primer |
| --- | --- | --- |
| SSX2 wild type (AA1-188) | ATGAACGGAGACGACGC | TCACTCGTCATCTTCCTCAGG |
| AA24-188 | GCCTTCGATGATATTGCCAAATAC | TCACTCGTCATCTTCCTCAGG |
| AA62-188 | GGTTTCAAGGCCACCCTC | TCACTCGTCATCTTCCTCAGG |
| AA1-154 | ATGAACGGAGACGACGC | TCATCTCTCGTGAATCTTCTCAGAG |
| AA62-154 | GGTTTCAAGGCCACCCTC | TCATCTCTCGTGAATCTTCTCAGAG |
| AA155-188 | TCTGGACCCAAAAGGGG | TCACTCGTCATCTTCCTCAGG |
| mutK158A * | GGACCCGCAAGGGGGGAACATGCC | CCCCCTTGCGGGTCCAGATCTCTCGT |
| mutR167A * | GAACATGCCTGGACCCACGCACTGCGTGAGAGAAAACAG | CTGTTTTCTCTCACGCAGTGCGTGGGTCCAGGCATGTTC |
| mutR169A * | CTGGACCCACAGACTGGCTGAGAGAAAACAGCTGGTGA | TCACCAGCTGTTTTCTCTCAGCCAGTCTGTGGGTCCAG |
| mutR171A, R172A * | ACCCACAGACTGCGTGAGGCAGCACAGCTGGTGATTTATGAAGAG | CTCTTCATAAATCACCAGCTGTGCTGCCTCACGCAGTCTGTGGGT |
| mutR167A, R169A, R171A, K172A * | GAACATGCCTGGACCCACGCACTGGCTGAGGCAGCACAGCTGGTGATTTATGAAGAG | CTCTTCATAAATCACCAGCTGTGCTGCCTCAGCCAGTGCGTGGGTCCAGGCATGTTC |
| mutS181A | ATGAACGGAGACGACGC | TCAGGGTCGGCGATCTCTTCATAAATCACCAGCT |
| del184-188 | ATGAACGGAGACGACGC | TCAAGGGTCGCTGATCTCTTCAT |

*Used together with wild type reverse and forward primers, respectively, to generate overlapping mutant PCR products, which were purified and assembled in a second PCR with only wild type primers.

**Primers for cloning SSX2 mutants into the pLVX-TetOne-puro plasmid using InFusion cloning**

| **SSX2 mutant** | **Forward primer** | **Reverse primer** |
| --- | --- | --- |
| SSX wild type (AA1-188) | CCCTCGTAAAGAATTCACCATGAACGGAGACGACGC | GAGGTGGTCTGGATCCTCACTCGTCATCTTCCTCAGG |
| Single nucleotide mutants | CCCTCGTAAAGAATTCACCATGAACGGAGACGACGC | GAGGTGGTCTGGATCCTCACTCGTCATCTTCCTCAGG |
| del184-188 | CCCTCGTAAAGAATTCACCATGAACGGAGACGACGC | GAGGTGGTCTGGATCCTCATCTCTCGTGAATCTTCTCAGAG |

**PRIMERS FOR SATELLITE PCR, RT-PCR AND CHIP-PCR**

**Genome primers for ChIP-PCR and qPCR expression analysis**

| **Target** | **Forward primer** | **Reverse primer** |
| --- | --- | --- |
| SATII* | ATCGAATGGAAATGAAAGGAGTCA | GACCATTGGATGATTGCAGTCA |
| SATIII** | AGT CCA TTC AAT GAT TCC ATT CCA GT | AAT CAT CAT CCA ACG GAA GCT AAT G |

The specificity of the primers were tested by gel electrophoresis in replicates (R1-R3) with and without DOX to induced SSX2 expression (see below). PCR conditions are provided in the Materials and Methods section:


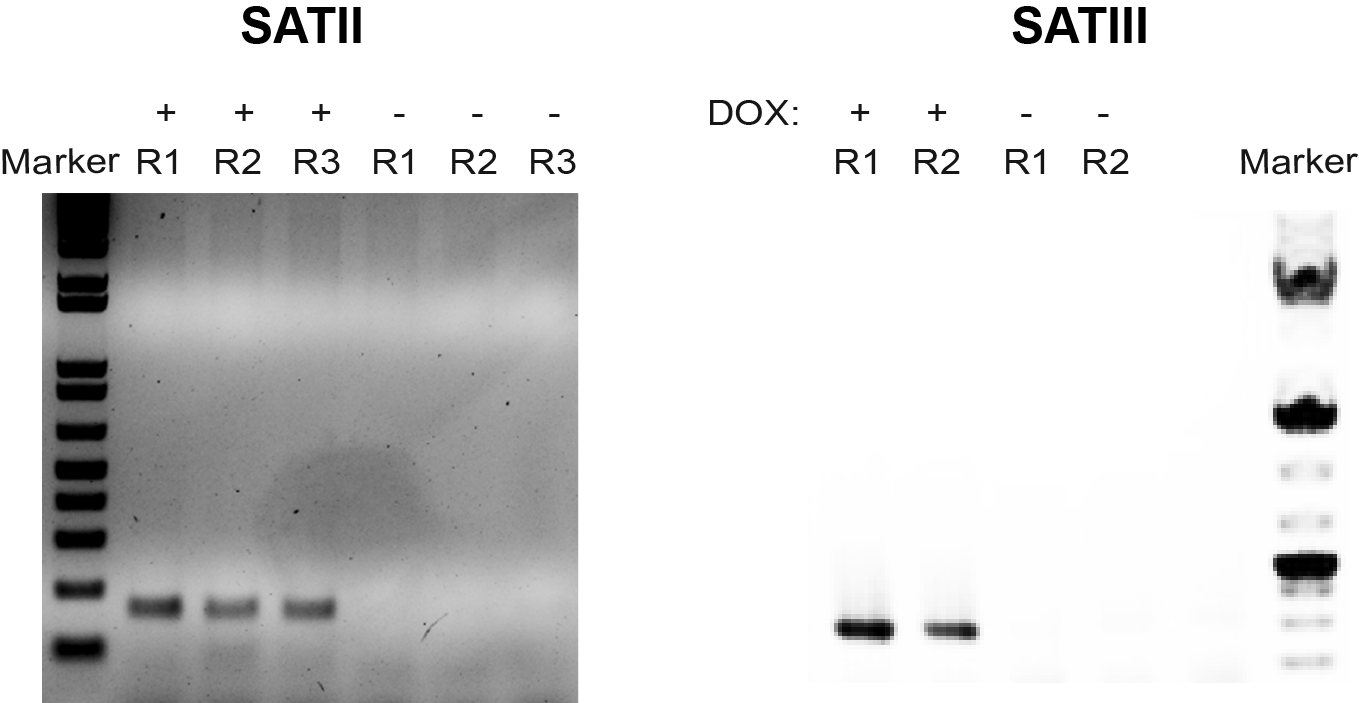


**Primers for SATII, SATIII and SAT- α reverse transcription**

| **Name** | **Sequence** |
| --- | --- |
| SATII sense* | GACCATTGGATGATTGCAGTCA |
| SATII antisense* | ATCGAATGGAAATGAAAGGAGTCA |
| SATIII sense** | AGTCCATTCAATGATTCCATTCCAGT |
| SATIII antisense** | AATCATCATCCAACGGAAGCTAATG |
| SAT- α sense+antisense*** | AAGGTCAATGGCAGAAAAGAA CAACGAAGGCCACAAGATGTC |

*SATII primers were designed to amplify a part of a published 1q12 SATII sequence (accession number: X72623.1). This structure shows high identity to pericentromeric heterochromatin structures on chromosome 1, 9 and 10.

**Adapted from Enukashvilly et al. *Cytogenet Genome Res*, 118, 42-54. The SATIII amplicon is contained in the BAC clone RP11-327C24 (genBank Acc. No. AC079934.3).

*** SAT- α primers were designed to amplify a part of a published SAT-α sequence (accession number: M26919.1) cooresponding to structures of the chromosome 1, 5 and 9 centromere.

**LNA GAPMER TARGET SEQUENCES**

| **Name** | **Sequence** |
| --- | --- |
| Negative control | AACACGTCCTATACGC |
| SATII-1 (sense) | CCATTCGATAATTCCG |
| SATII-2 (sense) | CGATTCCATTCAATTC |
| SATII-3 (sense) | CGATGACGATTCCATT |
| SATIII-1 (sense) | CGAATTGAAACGAATG |
| SATIII-2 (sense) | GATAATTCCATTCGT |
| SATIII-3 (antisense) | GAGTCCATTCAATGAT |
| SATIII-4 (antisense) | GAATGGAATCGTCATA |

**FISH PROBES**

| **Name** | **Probe** |
| --- | --- |
| 1q12 SATIII DNA FISH | LPE 001R/G, (Cytocell, Cambridge, UK) |
| 1q12 SATII RNA FISH (5’ and 3’ 5TYE563 label)* | ATTCATTCGATGACGATTCCA |

*The SATII probe was designed to target transcripts from the published 1q12 SATII sequence (accession number: X72623.1). This structure shows high identity to pericentromeric heterochromatin structures on chromosome 1, 9 and 10.

**PROBES FOR NORTHERN BLOTTING**

| **Name** | **Sequence** |
| --- | --- |
| SATII antisense probe | AGGAGTCACCATCTAATGGAATTGCATGGAATCATCATCA |
| SATII sense probe | TGATGATGATTCCATGCAATTCCATTAGATGGTGACTCCT |
| SATIII antisense probe | TCCATTCGAATCCATTCGATGATGAGTCCATCCATTTCAA |
| SATIII sense probe | GAATCGTCATAGAATGGAATCGAATGGATTCATTGAATGG |
| 5S probe | TGCTTAGCTTCCGAGATCAGACGAGATCGGGCGCGTTCA |

**DNA FRAGMENTS**

**276-bp SATII sequence used for EMSA**

This sequence is derived from X72623.1 and is repeated with small variations multiple times at the 1q12 pericentromeric heterochromatin domain. This structure shows high identity to pericentromeric heterochromatin structures on chromosome 1, 9 and 10.

GGACTCGAATGCAATCATCATCGAATGGAATGGAATGGAATCATCGAATGGACTCGAATGGATGGAACATTGAATCGAATGGAATCATCAATCGGATGGAAACGAATGGAATCATCATCGAATGGAAATGAAAGGAGTCATCATCTAATGGAATTGCATGGAATCATCATAAAATGGAATCGAATGGAATCAACATCAAATGGAATCAAATGGAATCATTGAACGGAATTGAATGGAATCGTCATCGAATGAATTGACTGCAATCATCCAATGGTC

**Sequencing of satellite II transcripts from A375 cells with SSX2 expression**

SATII sense: ATCGAATGGAAATGAAAGGAGTCACCATCTAATGGAATTGCATGGAATCATCATCAAATGGAATCGAATGGAATCAACATCAAATGGAATCAAATGGAATCATTGAACGGAATTGAATGGAGTCGTCATCGAATGAATTGACTGCAATCATCCAATGGTC

SATII antisense:

GACCATTGGATGATTGCAGTCAATTCATTCGATGACGATTCCATTCAATTCCGTTCAATGATTCCATTTGATACCATTTGATGTTGATTCCATTCGATTCCATTTTATGATGATTCCATGCAAGTCCATTAGATGATGACTCCTTTCATTTCCATTCGAT

These sequences are complimentary and exhibit high identity to multiple pericentromeric satellite DNA structures on chromosome 1 and 10.
