# Supplementary material for: Remodeling and destabilization of chromosome 1 pericentromeric heterochromatin by SSX proteins
Source: Nucleic Acids Res. 2019 May 22;47(13):6668–84. doi: 10.1093/nar/gkz396 (PMC6648343; doi:10.1093/nar/gkz396)
Supplement: gkz396_Supplemental_Files [file gkz396_supplemental_files.zip › Supplementary data.docx]

**SUPPLEMENTARY FIGURES**

**Figure S1. Alignment of SSX family members.** Human SSX proteins share 73-92% identity and contain a Krüppel associated box (KRAB; green) and an SSX repression domain = (SSXRD; blue). An exception is the alternative variant of SSX2, which lacks the SSXRD due to alternative splicing. Other isoforms of SSX proteins may exist.

**SSX1** MNGDDTFAKRPRDDAKASEKRSKAFDDIATYFSKKEWKKMKYSEKISYVYMKRNYKAMTKLGFKVTLPPF 70

**SSX2**  MNGDDAFARRPTVGAQIPEKIQKAFDDIAKYFSKEEWEKMKASEKIFYVYMKRKYEAMTKLGFKATLPPF 70

**SSX3** MNGDDTFARRPTVGAQIPEKIQKAFDDIAKYFSKEEWEKMKVSEKIVYVYMKRKYEAMTKLGFKAILPSF 70

**SSX4** MNGDDAFARRPRDDAQISEKLRKAFDDIAKYFSKKEWEKMKSSEKIVYVYMKLNYEVMTKLGFKVTLPPF 70

**SSX5** MNGDDAFVRRPRVGSQIPQKMQKAFDDIAKYFSEKEWEKMKASEKIIYVYMKRKYEAMTKLGFKATLPPF 70

**SSX6** MNGDDAFAKRPRDDAKASEKRSKAFDDIAKYFSKEEWEKMKFSEKISCVHMKRKYEAMTKLGFNVTLSLF 70

**SSX7** MNGDDAFARRPRAGAQIPEKIQKSFDDIAKYFSKKEWEKMKSLEKISYVYMKRKYEAMTKLGFKATLPPF 70

**SSX8** MNGDDAFAKRPRDDDKASEKRSKAFNDIATYFSKKEWEKMKYSEKISYVYMKRNYEAMTKLGFNVTLPPF 70

**SSX9** MNGDDAFARRPRAGSQIPEKIQKAFDDIAKYFSKKEWEKMKSSEKIIYVYMKRKYEAMTKLGFKATLPPF 70

**SSX2alt**MNGDDAFARRPRDGAQIPEKIQKAFDDIAKYFSKKEWEKMKSSEKISYVYMKRKYEAMTKLGFKVTLPPF 70

**SSX1** MCNKQATDFQGNDFDNDHNRRIQVEHPQMTFGRLHRIIPKIMPKKPAEDENDSKGVSEASGPQNDGKQLH 140

**SSX2**  MCNKRAEDFQGNDLDNDPNRGNQVERPQMTFGRLQGISPKIMPKKPAEEGNDSEEVPEASGPQNDGKELC 140

**SSX3** MRNKRVTDFQGNDFDNDPNRGNQVQRPQMTFGRLQGIFPKIMPKKPAEEGNVSKEVPEASGPQNDGKQLC 140

**SSX4** MRSKRAADFHGNDFGNDRNHRNQVERPQMTFGSLQRIFPKIMPKKPAEEENGLKEVPEASGPQNDGKQLC 140

**SSX5** MRNKRVADFQGNDFDNDPNRGNQVEHPQMTFGRLQGIFPKITPEKPAEEGNDSKGVPEASGPQNNGKQLR 140

**SSX6** MRNKRATDSQRNDSDNDRNRGNEVERPQMTFGRLQRIIPKIMPEKPAEEGSDSKGVPEASGPQNDGKKLC 140

**SSX7** MHNTGATDLQGNDFDNDRNQGNQVERPQMTFCRLQRIFPKIMPKKPAEEGNDSKGVPEASGSQNDGKHLC 140

**SSX8** MCNKQATDFQGNYFDNDRNRRIQVERPQMTFGRLQRIIPKIMPKKPAEEGNDSKGVSEASGPQNDGKQLA 140

**SSX9** MCNTGATDLQGNDFDNDRNHRNQVERSQMTFGRLQGIFPKIMPKKPAEVGNDSKEVPEASGLQNDGKQLC 140

**SSX2alt**MRNKRATDFQGNDFDNDRNRGNQVERPQMTFGRLQRIFPKIMPKKPAEEGNDSKGVPEASGPQNDGKQLC 140

**SSX1** PPGKANISEKINKRSGPKRGKHAWTHRLRERKQLVIYEEISDPEEDDE 188 (NCBI acc. CAA60110.1)

**SSX2** PPGKPTTSEKIHERSGPKRGEHAWTHRLRERKQLVIYEEISDPEEDDE 188 (NCBI acc. CAA60111.1)

**SSX3** PPGKPTTSEKINMISGPKRGEHAWTHRLRERKQLVIYEEISDPEEDDE 188 (NCBI acc. CAA05819.1)

**SSX4** PPGNPSTLEKINKTSGPKRGKHAWTHRLRERKQLVVYEEISDPEEDDE 188 (NCBI acc. CAA05820.1)

**SSX5** PSGKLNTSEKVNKTSGPKRGKHAWTHRVRERKQLVIYEEISDPPEDDE 188 (NCBI acc. CAA05821.1)

**SSX6** PPGKASSSEKIHERSGPKRGKHAWTHRLRERKQLVIYEEISDPEEDDK 188 (NCBI acc. CAA40525.1)

**SSX7** PPGKPSTSEKINKTSGPKRGKHAWTHRLRERKQLVIYEEISDPEEDDE 188 (NCBI acc. NP775494.1)

**SSX8** P-GKANTSEKINKRSGPKRGRHAWTHRLRERNQLVIYEEIRDPEEDDE 187 (NCBI acc. Q7RTT4.2)

**SSX9** PPGKPTTSEKINKASGPKRGKHAWTHRLRERKQLVIYEEISDPEEDDE 188 (NCBI acc. Q7RTT3.1)

**SSX2alt**PPGKPNTSEKINKRSGNREAQEKEERRGTAHRWSSQNTHNIGRFSLSTSMGAVHGTPKTITHNRDPKGGN 210

**SSX2alt**MPGPTDCVRENSW 223 (NCBI acc. AIC49797.1)

**Figure S2. SSX2 depletes PcG body chromatin domains.** (**A**) SSX2 target Pc bodies in A375 melanoma cells as demonstrated by the specific overlap with core PRC1 proteins BMI1 and RING1B and PcG-associated chromatin modifications H2AK119ub and H3K27me3. (**B**) SSX2 was expressed under a doxycycline- (DOX) inducible promoter in A375 cells for 24 hours and shown to disintegrate Pc body chromatin domains. (**C**) Quantification of results from panel B. Number of hours with SSX2 expression is indicated. A two-tailed students t-test was used for statistical analysis. Data represent the mean ± SD for three biological replicates. More than 100 cells were analyzed per sample. Scale bars = 10 µm.

**
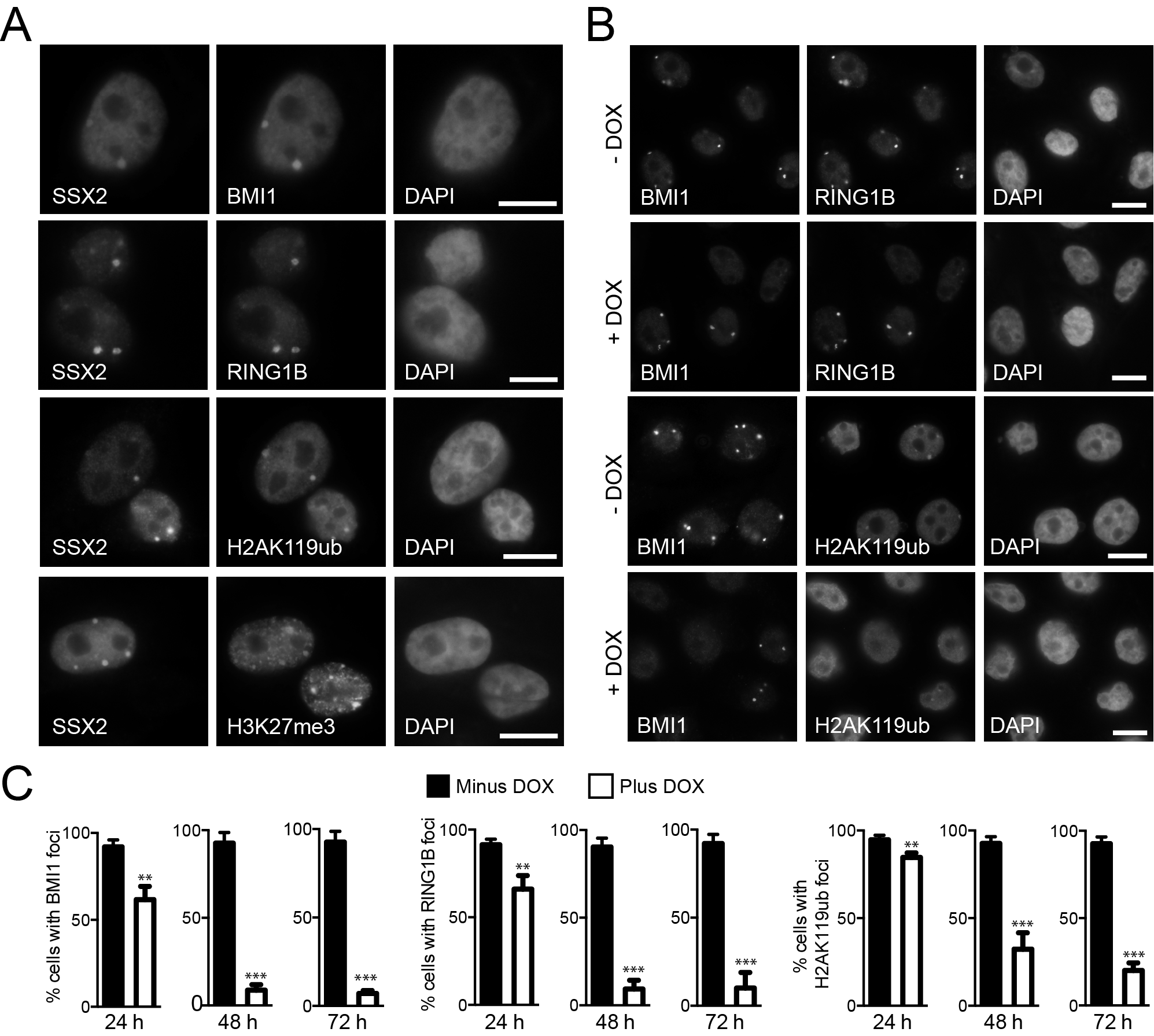
**

**Figure S3. PcG body targeting of SSX2 family members and alternative splice variants.** A375 melanoma cells were transfected with pLX304 expression plasmids obtained from the Harvard Medical School plasmid repository encoding different SSX family members with a V5 tag. Cells were stained with anti-V5 and anti-BMI1 antibodies. SSX1, SSX2, SSX3 and SSX4 were demonstrated to target PcG bodies, which was not the case for the alternative variant of SSX2 (SSX2 alt) lacking the SSXRD due to alternative splicing.


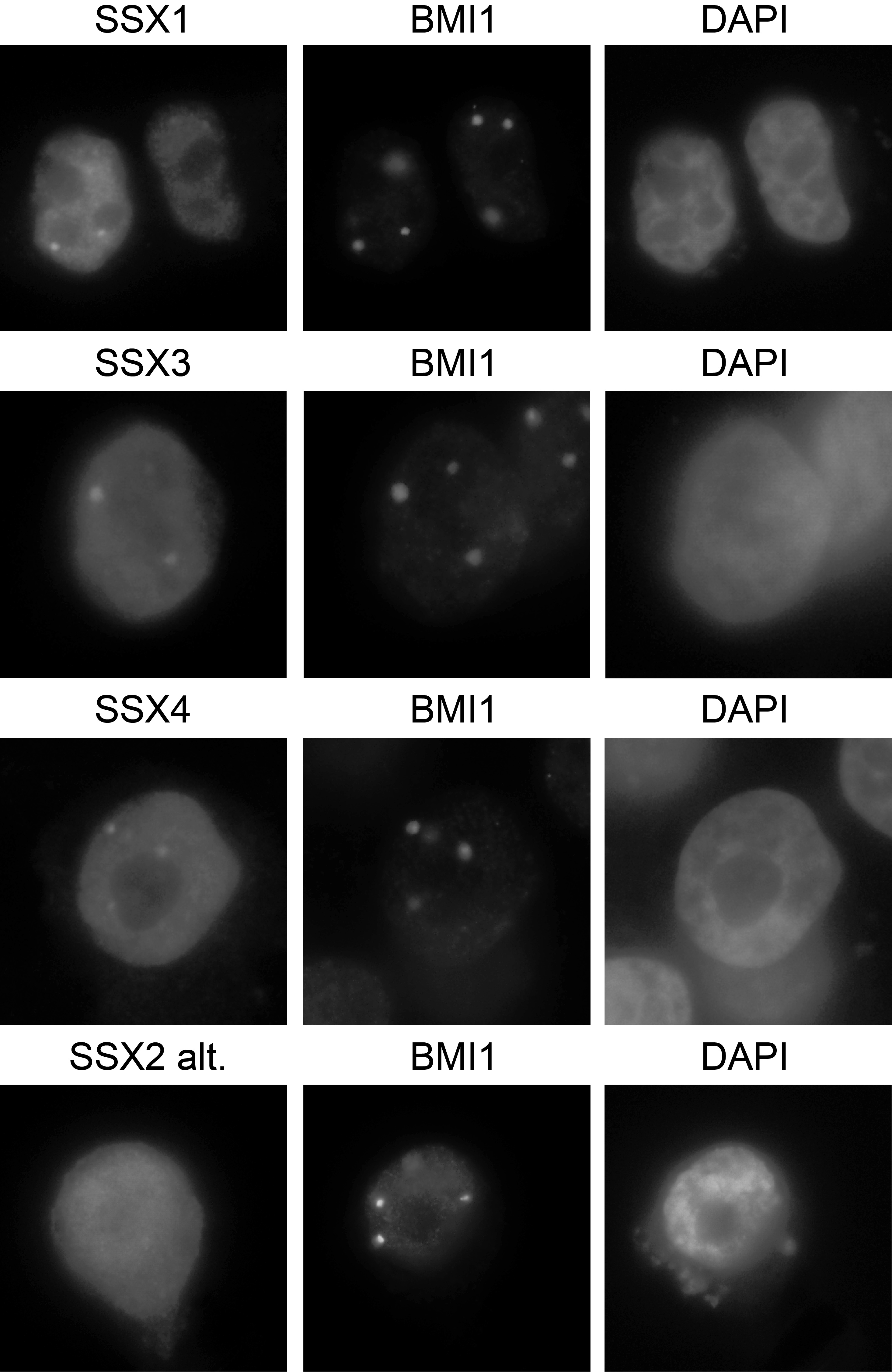


**Figure S4. ChIP-PCR analysis of BMI1** occupancy at chromosome 1 centromeric α-satellite DNA in A375 cells with doxycycline (DOX)-induced SSX2 expression. As a negative control, IgG was used.

**Figure S5. SSX2 distends 1q12 satellite DNA in FM28 melanoma cells.** FM28 melanoma cells were transduced with lentivirus carrying the pCDH-CMV-Puro-GFP-SSX2 expression plasmid. After 72 hours the cells were stained with a combined BMI1 immunostaining-1q12 satellite III FISH protocol. Scale bars = 10 µm.


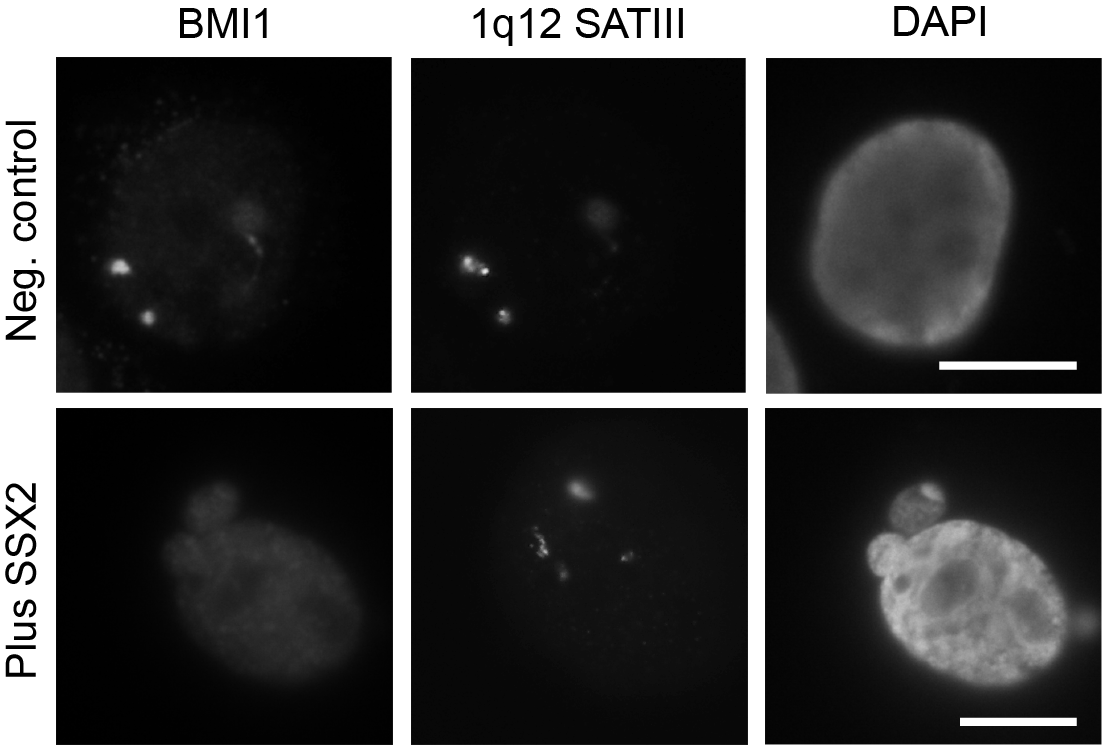


**Figure S6. SSX2 expression does not alter the structure of centromers.** SSX2 expression was induced with doxycycline (plus DOX) in A375 melanoma cells and after 48 hours the centromeric protein CENP-B was stained. Scale bars = 10 µm.

**
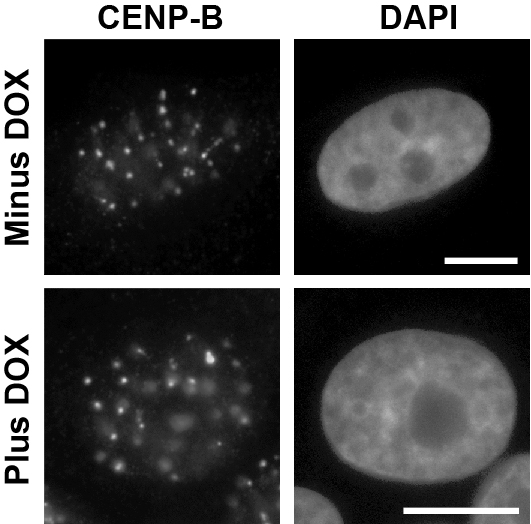
**

**Figure S7.** **Dose-dependent depletion of PcG bodies and induction of genomic instability by SSX2 in A375 melanoma cells.** Induction of increasing levels of SSX2 expression with increasing levels of doxycycline (DOX) in A375 melanoma cells. SSX2 exhibits a concentration dependent targeting and depletion of PcG bodies that correlates with formation of micronuclei. Scale bars = 50 µm.

**
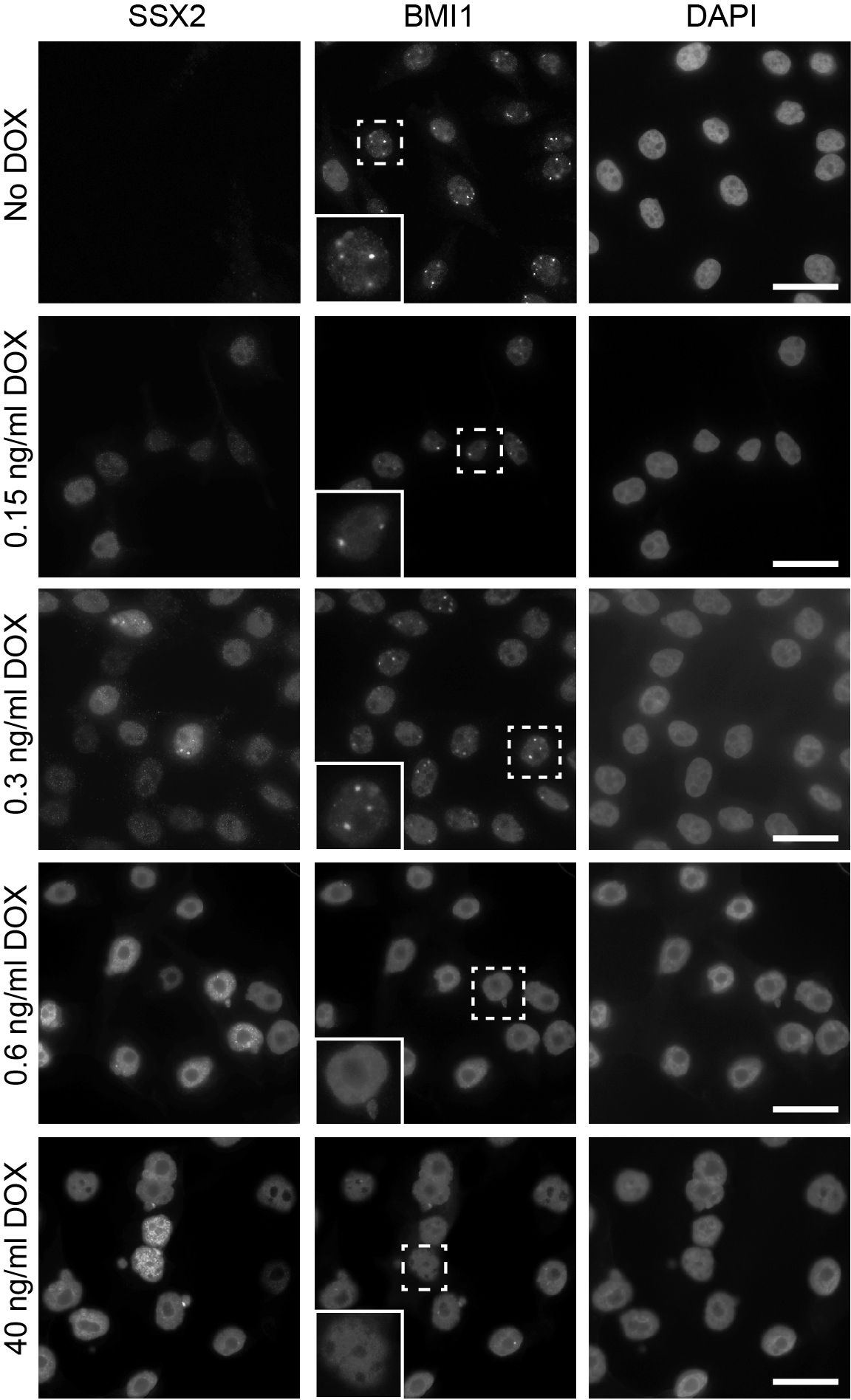
**

**Figure S8. Bisulfite sequencing of a 1q12 satellite III locus in A375 and MCF7 cells.** Non-converted (i.e. methylated) CpG sites are highlighted in red.

A375-1

AATTCCATTCCATTCCATTCAATAATTCCATTCCAAACCATTCAATAATTCCATCTAACTCCATTCAATAATTCCATTCCATTCCATTCAATAATTCCATTTAATTCCATTTAATATTAATTCCATTCAATTCCATTTTATAATAATTCCATACAATTCCATTAAATAATAACCCCTTTCATTTCCA

A375-2

AATTCCATTCCATTCCATTCGATAATTCCATCTAATTCCATTCAATAAATCCATTCAATTCCATTCTATAACAATTCCATTCATTTCCATTTGATGATGATTCCATTCAATTCCATTTGATAATGATTCCATACAATTCCATTAAATAATAACCCCTTTCATTTCCA

A375-3

AATTCCATTCCATTCCATTCGATAATTCTATCTAATTCCATTCAATAAATCCATTCAATTCCATTCTATAACAATTCCATTCATTTCCATCTAATAATAATTCCATTTAATTCCATTTAATATTAATTCCATTCAATTCCATTTTATAATAATTCCATACAATTACATTAAATAATAACCCCTTTCATTTCCA

A375-4

AATTCCATTCCATTCCATTCGATAATTCCATTCCATTCCATTCAATAATTCCATTTAATTCCATTTAATATTAATTCCATTCAATTCCATTTTATAATAATTCCATACAATTCCATTAAATAATAACCCCTTTCATTTCCA

A375-5

AATTCCATTCCATTCCATTCAATAATTCCATTTAATTCCATTTAATATTAATTCCATTCAATTCCATTTTATAATAATTCCATACAATTCCATTAAATAATAACCCCTTTCATTTCCA

A375-6

AATTCCATTCCATTCCATTCAATAATTCCATCTAATTCCATTCAATAAATCCATTCAATTCCATTTTATAATAATTCCATACAATTCCATTAAATAATAACCCCTTTCATTTCCA

A375-7

AATTCCATTCCATTCCATTCAATAATTCCATTAAATTCCATTTAATATTAATTCCATTCAATTCCATTTAATAATAATTCCATACAATTCCATTAAATAATAACCCCTTTCATTTCCA

A375-8

AATTCCATTCCATTCCATTCGATAATTCCATTCCATTCCATTTAAAAATAATTCCATTCAAAACCATTCAATAATTACATTCAATTCATTCAATAACAATTCCATTCAATTCCGTTCAATAATTCCATTTAATTCCATTTAATATTGATTCCATTCGATTCCATTTTATAATAATTCCATACAATTCCATTAAATAATAACCCCTTTCATTTCCA

A375-9

AATTCCATTCCATTCCATTCAATAATAATTCCATTCAATTCTATACAATAATTCCATTCCATTCCATTCAATAACTCTATCTAATTCCATTCAATAAATCCATTAAATTCCATTCTATAACAATTCCATTCATTTCCATCTAATAATAATTCCATTCAATTCCATTCAATAATTACACTTAATTCCATTTAATATTAATTCCATTCAATTCCATTTTATAATAATTCCATACAATTCCATTAAATAATAACCCCTTTCATTTCCA

MCF7-1

ATTCCATTCCATTCCATTCGATAATTCCATCTAATTCCATTCAATAAATCCATTCAATTCCATTCTATAACAATTCCATTCGATTCCATTTAATAATAATTCCATACGATTCCATTAAATAATAACCCCTTTCATTTCCA

MCF7-2

AATTCCATTCCATTCCATTCGATAATAATTACATTCAAATCCATTCGATAATTCCATTCCTTTCCATTAAAAAATAATTCCATTCAAAACCATTCAATAATTACATTCAATTCATTCGATAACGATTCCATTCAATTCTATTTAATTATTCCCTTAAATTCCATTTAATAATAATTCCATTCGATTCCATTTAATGATAATTCCATACGATTCCATTAAATAATAACCCCTTTCATTTCCA

MCF7-3

AATTCCATTCCATTCCATTCGATAATAATTACATTCGAATCCATTCAATAATTCCATTCCATTCCATTTAAAAATAATTCCACTCAAAACCATTCGATAATTACACTCAATTCATTCGATAACGATTCCATTCAATTCCATTCAATAATTCCATTTAATTCCATTTAATATTAATTCCATTCAATTCCATTTAATAATAATTCCATACAATTCCATTAAATAATAACCCCTTTCATTTCCA

MCF7-4

AATTCCATTCCATTCCATTCGATAATCCCATCTAATTCCATTCAATAAATCCATTCGATTACATTCTATAACGATTCCATTCAATTCTGTTCAATAATTCCATTAAATTCCATTTAATAAAAATTCCATTCAATTCCATTTAATAATAATTCCATACGATTCCATTAAATAATAACCCCTTTCATTTCCA

MCF7-5

AATTCCATTCCATTCCATTCGATAATTCCATTCCATTCCATTTAAAAATAATTCCATTCGAAACCATTCGATAATTACATTCAATTCATTCGATAACGATTCCATTAAATTCTATTCAATAATTCCATCAAATTCCATTTAATAATAATTCCATTCAATTCCATTTAATAATAATTCCATAAAATCCCATTAAATAATAACCCCTTTCATTTCCA

MCF7-6

AATTCCATTCCATTCCATTCGATAATTCCATCTAATTCCATTCAATAAATCCATTCAATTCCATTCTATAACGATTCCATTAAATTCCATTCAATAATTCCATTTAATTCCATTTAATATTAATTCCATTCGATTCCATTTTATAATAATTCCATACAATTCCATTAAATAATAACCCCTTTCATTTCCA

MCF7-7

AATTCCATTCCATTCCATTCGATAATTCCATCTAATTCCATTCAATAAATCCATTCGATTCCATTCTATAACGATTCCATTCATTTCCATCTAATAATAATTCCATTCGATTCCATTAAATAATAACCCCTTTCATTTCCA

**Figure S9. The effect of SSX2 point mutants on targeting and depletion of PcG bodies.** SSX2 mutants were expressed with a N-terminal GFP tag using the psDNA6.2-EmGFP plasmid (Thermo Fisher Scientific, Naerum, Denmark) in A375 melanoma cells and cells were stained for BMI1 PcG bodies. Scale bars = 50 µm.


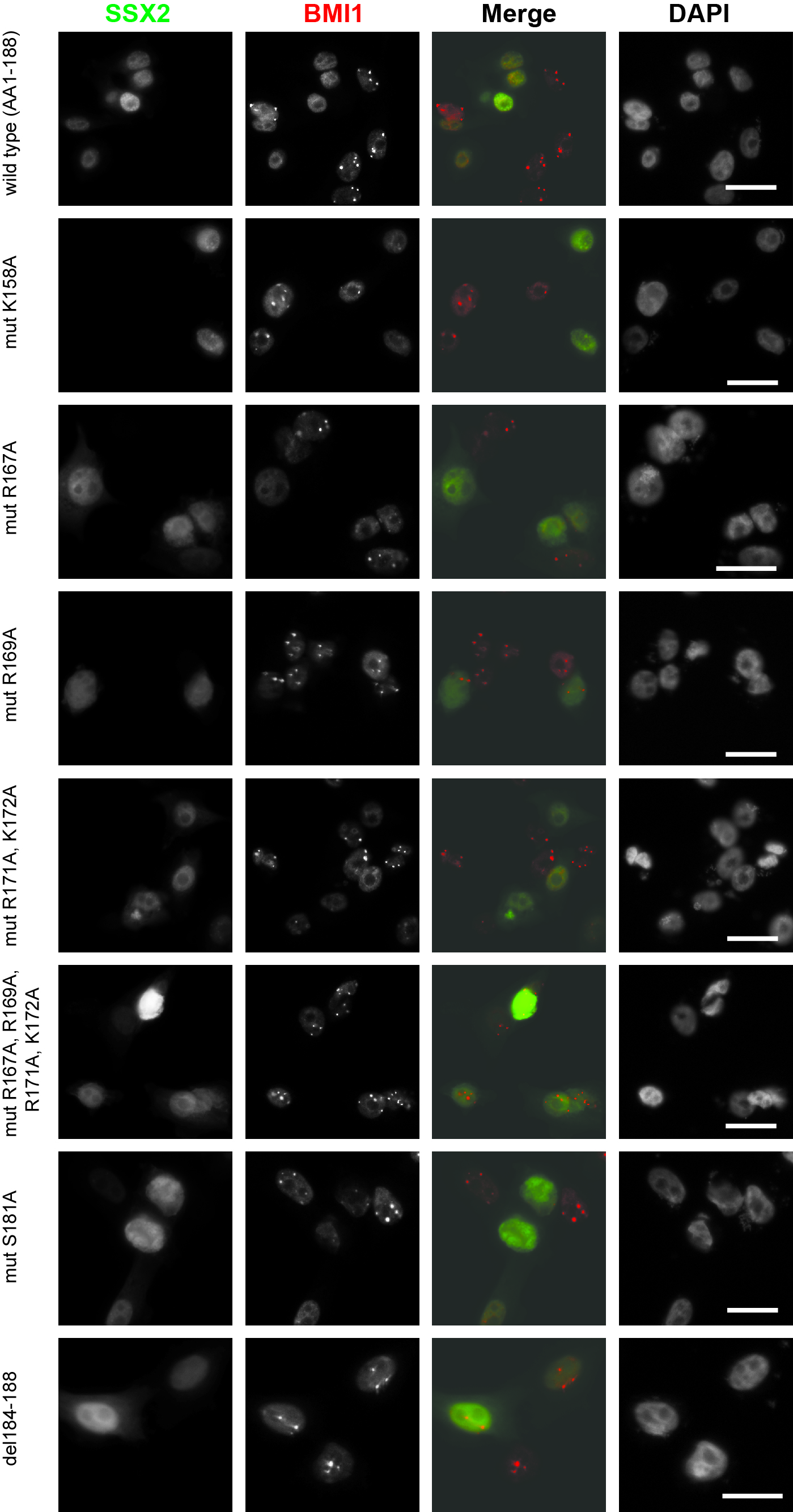


**Figure S10. Overview of the structural modulation of SSX2 using the The Phyre2 web portal made available by the Structural Bioinformatics Group, Imperial College London** (http://www.sbg.bio.ic.ac.uk/phyre2/html/page.cgi?id=index).

**
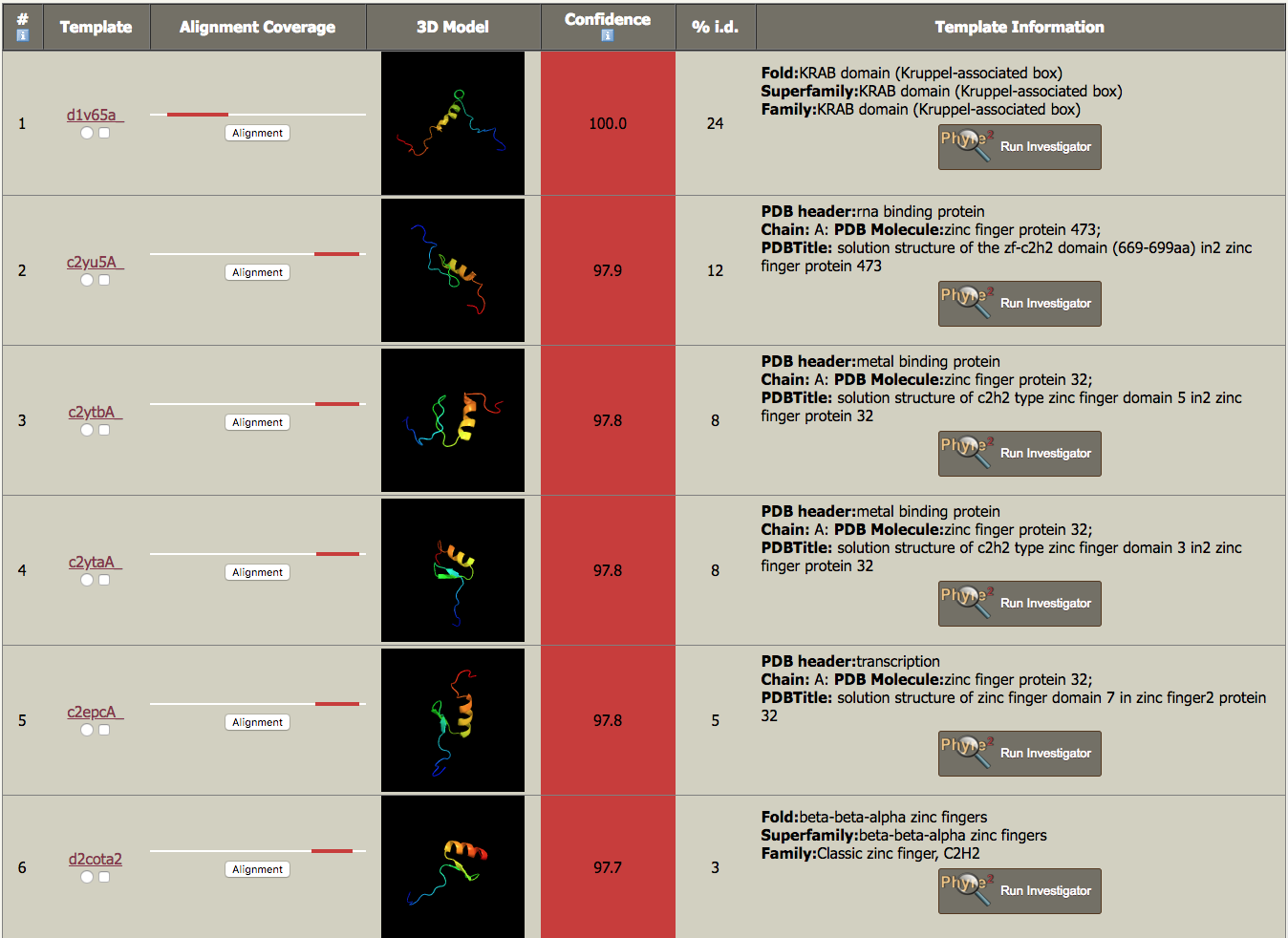
**

**Figure S11. The role of SSX interaction partners on SSX2-mediated PcG depletion and 1q12 PCH unfolding.** (**A**) ZBED1 was expressed in A375 cells with a V5 tag and demonstrated to localize to BMI1 PcG bodies using immunocytochemistry. (**B**) To examine the role of ZBED1 in SSX2-mediated PcG depletion we knocked down ZBED1 expression using shRNA delivered by lentivirus and quantified the effect on the presence of BMI1 PcG bodies in A375 cells. (**C-D**) The localization of two known SSX interactions partners, SSXIP (C) and KDM2B (D), was characterized by immunocytochemistry. Neither showed any colocalization with BMI-PCG bodies. Data represent the mean ± SD for three biological replicates. A two-sided t-test was used for statistical analysis (* < 0.05, ** < 0.001, *** < 0.0001). More than 100 cells were analyzed per sample. Scale bars = 10 µm.

**
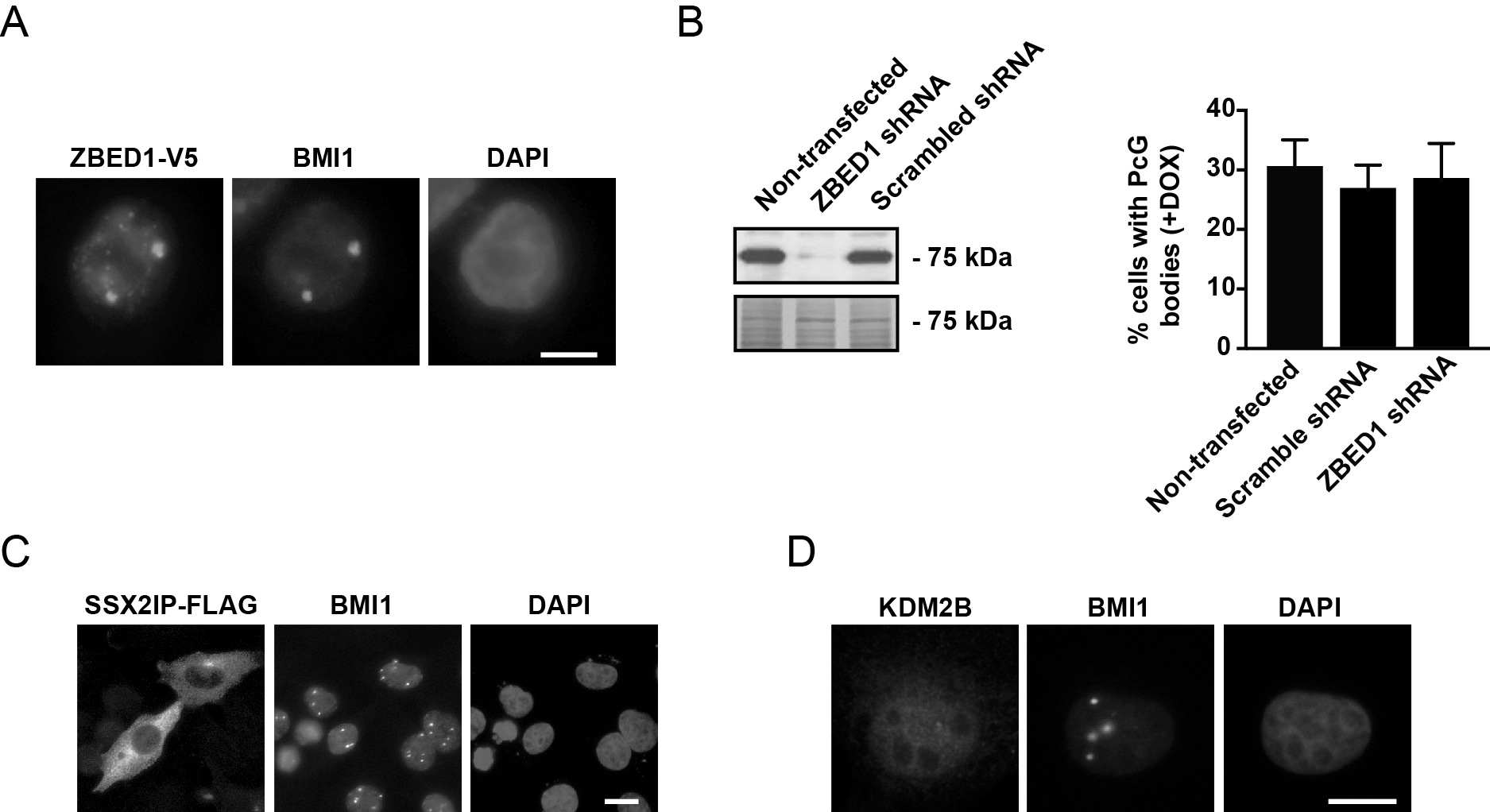
**

**Figure S12. SSX2 forms oligomers in vitro.** Recombinant SSX2 was crosslinked by glutaraldehyde and analyzed by Western blotting revealing potential dimers (*) and higher order oligomers (**).


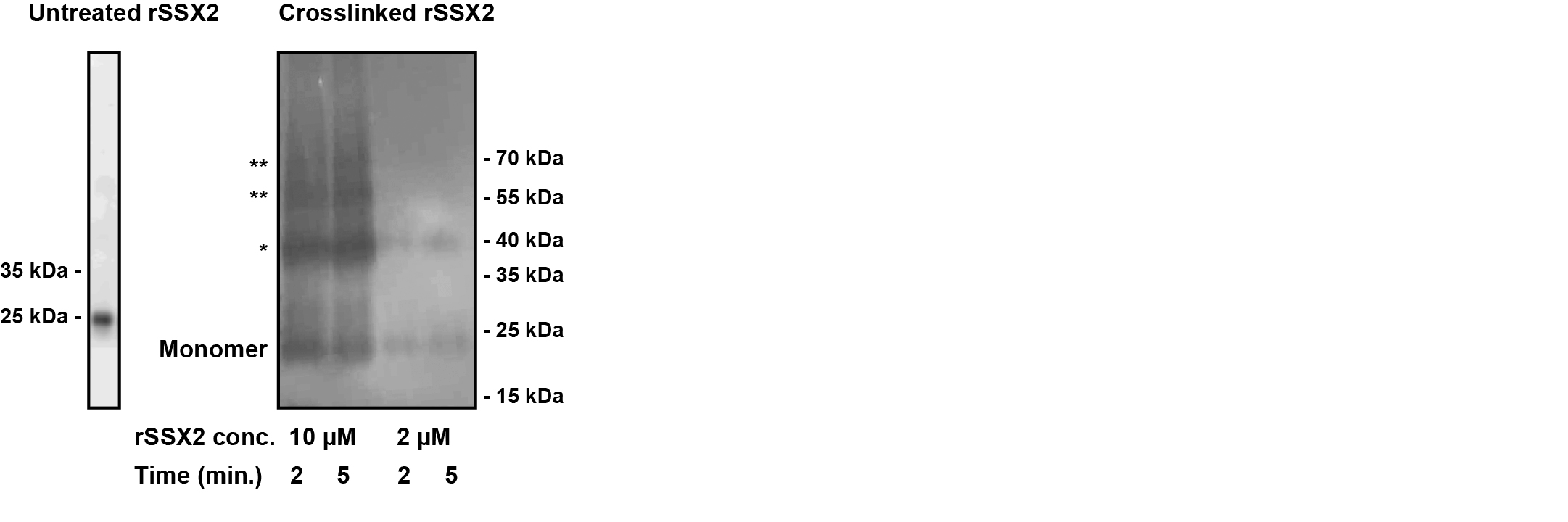


**Figure S13. SSX2 does not interact through the KRAB domain at PcG bodies.** Full-length SSX2 cannot recruit SSX2-AA1-154 (with N-terminal GFP-tag) to BMI1 PcG bodies, suggesting that SSX2 proteins do not interact through their KRAB domain at these structures.

**
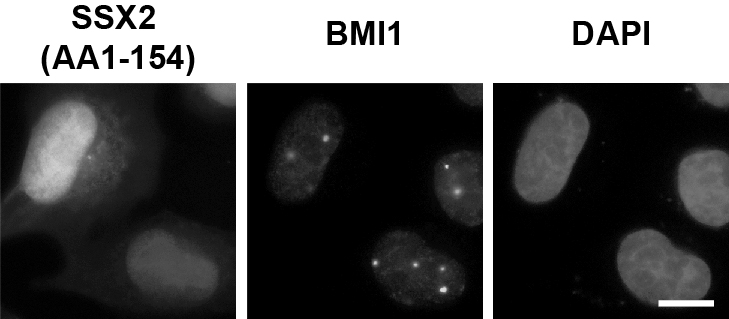
**

**Figure S14. 1q12 PCH transcripts does not affect stability of PcG bodies.** (**A**) A375 cells were transfected with SATII-targeting LNA GapmeRs. Reduced levels of SATII transcripts were confirmed by RT-qPCR 72 hours after transfection. (**B**) A375 cells were transfected with SATIII-targeting LNA GapmeRs with high effect on cell proliferation/viability 72 hours after transfection, preventing evaluation of the reduction in transcript levels. A crystal violet cell staining assay was used for cell quantification. (**C**) Neither SATII nor SATIII LNA GapmeRs affected the SSX2-mediated disintegration of PcG bodies. (**D**) Quantification of the effect of SATII LNA Gapmer transfection on SSX2-mediated disintegration of PcG bodies. (**E**) Quantification of the effect of SATIII LNA Gapmer transfection on SSX2-mediated disintegration of PcG bodies. (**F**) 160-bp 1q12 SATII sense and antisense transcripts were cloned from A375 cells with SSX2 expression and expressed under the control of a CMV promoter in A375 cells without SSX2 expression. (**G**) The 160-bp SATII transcripts did not affect the stability of BMI PcG bodies or induce genomic instability (i.e. formation of micronuclei). (**H**) RNA fish with a SATII probe shows overlap between PcG bodies and SATII transcripts. Data represent the mean ± SD for three biological replicates. A two-sided t-test was used for statistical analysis (* < 0.05, ** < 0.001, *** < 0.0001). More than 100 cells were analyzed per sample. Scale bars = 10 µm.

**
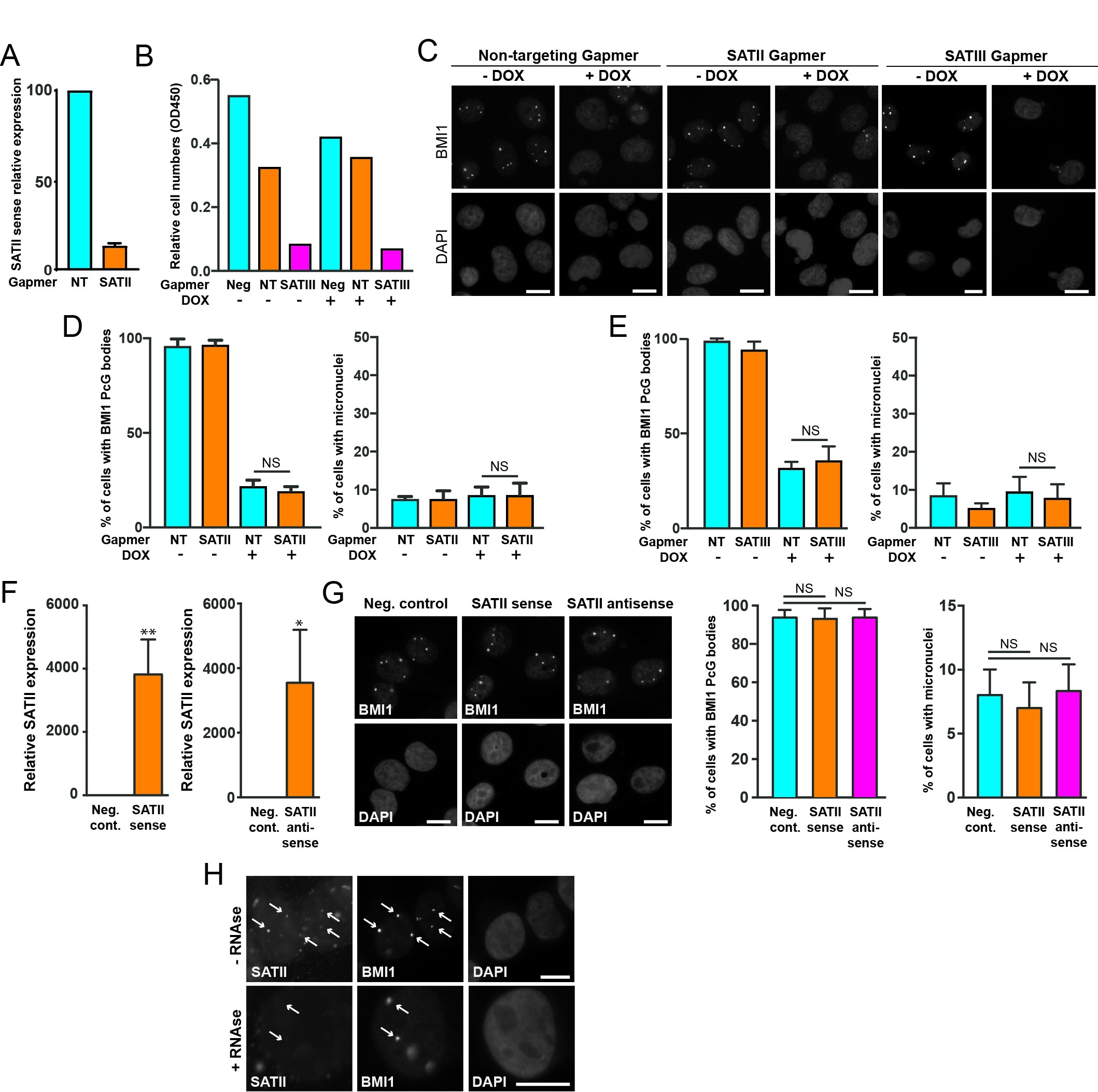
**

**Figure S15. SSX2 localize to PcG bodies 6 hours after induction of SSX2 expression in A375 cells with doxycycline.** SSX2 expression was induced by the addition of doxycycline and 6 hours later the cells were fixed and stained for SSX2 and BMI1. Scale bars = 10 µm

**
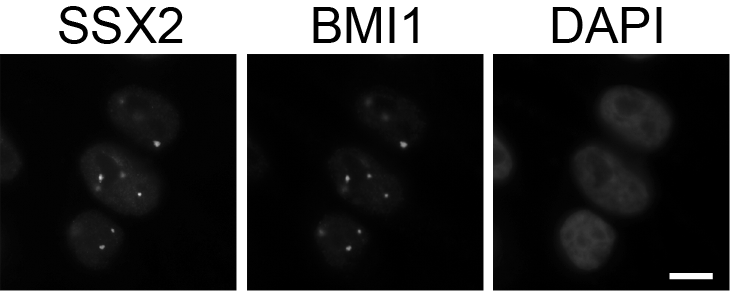
**
